# Qualitative and quantitative analyses of artificial intelligence ethics in education using VOSviewer and CitNetExplorer

**DOI:** 10.3389/fpsyg.2023.1061778

**Published:** 2023-03-09

**Authors:** Liheng Yu, Zhonggen Yu

**Affiliations:** ^1^School of Engineering, University of Birmingham, Edgbaston, Birmingham, United Kingdom; ^2^Faculty of Foreign Studies, Beijing Language and Culture University, Beijing, China

**Keywords:** artificial intelligence, ethics, bibliometric analysis, VOSviewer, CitNetExplorer

## Abstract

The new decade has been witnessing the wide acceptance of artificial intelligence (AI) in education, followed by serious concerns about its ethics. This study examined the essence and principles of AI ethics used in education, as well as the bibliometric analysis of AI ethics for educational purposes. The clustering techniques of VOSviewer (*n* = 880) led the author to reveal the top 10 authors, sources, organizations, and countries in the research of AI ethics in education. The analysis of clustering solution through CitNetExplorer (*n* = 841) concluded that the essence of AI ethics for educational purposes included deontology, utilitarianism, and virtue, while the principles of AI ethics in education included transparency, justice, fairness, equity, non-maleficence, responsibility, and privacy. Future research could consider the influence of AI interpretability on AI ethics in education because the ability to interpret the AI decisions could help judge whether the decision is consistent with ethical criteria.

## Introduction

Artificial intelligence (AI) was defined by Dodigovic (2007, p. 100) as the technology imitating human behaviors, which was an interdisciplinary inquiry aiming to perceive the way human brain worked and integrate human working mechanisms into technologies. AI has been widely adopted in various fields such as healthcare, transportation, communication, and education. AI could facilitate distance or seamless education by enhancing the flexibility, interactivity, and personalization of education, where teachers could be released from heavy tasks of scoring assignments (Zawacki-Richter et al., 2019). AI could also improve learning outcomes and facilitate educational procedures by providing great convenience for teachers and improve learning efficiency for students. Although AI has made enormous strides and been widely adopted in education, it has brought about a variety of issues, among which ethics of AI used in education is a knowledge gap to be filled.

The AI ethical issue in education has become an indispensable concern with the rapid advancement of AI technologies. The use of AI in education could bring about not only the pedagogical issues but also the ethical concerns (Zawacki-Richter et al., 2019). The development of AI has led educational styles to experience great changes, where AI has become an indispensable element and the ethical issue in AI-based education has also caught researchers’ attention (Bozkurt et al., 2021). The dramatically enhanced competitiveness of AI may pose a threat to human beings who may be dwarfed by the powerful AI technologies. How to strike a balance between humans and AI may be an urgent measure for researchers to take.

The use of AI for educational purposes has triggered the revolution of education since AI was extended to the educational field (Holmes, 2019). Ethics of the use of AI in education, although an essential aspect in the development of AI, has been disregarded in educational research, where ethical codes or guidelines with ethical principles are still scanty (Bozkurt et al., 2021). Until now, a wide gap has appeared between ethics research and the adoption of AI in education. It is thus meaningful to complement this missing link by examining the ethics of AI used in education through both qualitative and quantitative analyses.

Several researchers examined the ethics of AI used in education. AI, in education, is mainly used for adaptive system design, evaluation, individualization, monitoring, and assistance. As for the field of ethics of AI in education, relatively fewer critical analyses of challenges and risks were explored and ethics in the relation between AI and education needs to be further discussed in terms of theories and practices (Zawacki-Richter et al., 2019). Other studied themes included the ethical issue in STEM education (Lin et al., 2021), governance of AI (Jia, 2020), AI ethics in spiritual education (Tan, 2020), and ethical elements of AI (Wamba et al., 2021).

Students could learn ethics of AI as a subject, which could guide designers and developers to design and innovate AI technologies (Burton et al., 2017). As a subject, ethics aims to identify the way where the world should be perceived and what behaviors people should conduct. Many conceptions regarding ethics have risen, where basic questions about the world are proposed and ethical challenges are described. However, the conceptions of ethics rely on discussion and argumentation. When confronted with an ethical issue, academicians and learners gather to make judgments to establish norms for the problems which tend to undergo complicated interactions and negotiations.

Both opportunities and challenges of the use of AI have been explored. Floridi et al. (2018) formulated four core opportunities of the use of AI, i.e., human self-realization, human agency enhancement, societal capability increase, and societal cohesion cultivation. They were also considered the possibility of AI to be underused. By contrast, AI technologies could be abused if they devaluated human skills, removed human responsibility, reduced human control, or decreased human self-determination. By combining both opportunities and challenges in the use of AI, human beings could keep AI under control while facilitating the societal development. In this way, humans could strike a balance between AI technological development and socially harmonious formation.

Ethical issues regarding the use of AI in education posed great challenges to both researchers and practitioners (Dignum, 2021). Data containing personal information might be easily revealed or misused. Games based on AI technologies might be abused, which might hamper the learning outcomes (Zhai et al., 2021). UNESCO expressed its concern about ethical issues such as educational equity, inclusiveness, data security, privacy protection, and transparency of data use and collection (Pedró et al., 2019). Individual privacy was a serious concern in intelligent agent-assisted online learning (Yu et al., 2022). The intelligent agent could automatically retrieve private information such as learning strategies, styles, and performances. Prejudice against students who performed poorly might thus arise among teachers, negatively influencing the equality of education (Li, 2007, p. 327). This study, aiming to examine ethics of AI in education, is thus meaningful and worthy.

Considering the existing and increasingly serious ethical tensions (Wang et al., 2019) in AI used in education, it is necessary to define specific ethical guidelines and principles and establish ethical policies and limitations to the way how AI could adopt human-produced data (Goksel and Bozkurt, 2019). Ethics for AI used in education is expecting further exploration to keep pace with the rapid advancement of AI technologies integrated into education (Nye, 2016). This study will try to combine qualitative with quantitative research methods using both VOSviewer and CitNetExplorer. It will firstly review the studies on ethics of AI in education, then analyze the clustering solutions, and qualitatively examine the ethical essence and principles based on the included studies and participants’ opinions retrieved from a questionnaire survey.

In recent years, several review studies have been conducted to study AI applications in the education field. A systematic review explored four areas in AI ethics in education: overview and prediction, evaluation, system adaptation, and tutoring systems. Another review study revealed the scanty studies on challenges of AI ethics, as well as weak connections between theoretical and practical explorations (Zawacki-Richter et al., 2019). An interview with Yi Zeng, an expert in at the Institute of Automation, Chinese Academy of Sciences, revealed several ways to protect personal privacies and national security, and methods to cultivate proper human behaviors and avoid unethical use of AI technologies (Jia, 2020). A comparison between Confucius and Digital Confucius teachers revealed that human teachers could be replaced with AI partners in moral education and AI partners were also limited to knowledge transmittance and ethical issues (Tan, 2020). A bibliometric analysis explored the intellectual development, social changes, economic empowerment, conceptual structure of AI research, security, and justice (Wamba et al., 2021). However, it did not delve into the essence and principles of AI ethics in education.

The novelty of this study is to investigate the AI ethics through both VOSviewer and CitNetExplorer. This study aims to address the research questions including the publication trend, top authors, sources, organizations, and countries, the essences and principles of AI ethics in education. Firstly, this study will attempt to reveal the trend of publications of ethics in the use of AI in education using the techniques embedded in the online database “Web of Science.” Researchers selected the Core Collection of Web of Science because this online database is made of many high-quality publications and numerous online databases: Science Citation Index Expanded --2013-present, Social Sciences Citation Index--2008-present, Arts & Humanities Citation Index --2008-present, Emerging Sources Citation Index--2017-present, Current Chemical Reactions--1985-present, and Index Chemicus (IC)--1993-present. Secondly, the study will reveal the top authors, sources, organizations, and countries using VOSviewer. Finally, researchers will use the longest path in CitNetExplorer to investigate the essence and principles of ethics of AI used in education.

## Methods

This study involved three steps by using both qualitative and quantitative research methods. Firstly, we conducted this study using the framework of the Preferred Reporting Items for Systematic Reviews and Meta-Analyses (PRISMA; Moher et al., 2015), which included 17 items to direct the research process. The ethic committee waived the registry of this study since it does not violate any ethical codes. Besides, this study also conducted bibliometric analyses using VOSviewer and a qualitative analysis using CitNetExplorer. Both CitNetExplorer and VOSviewer are combined to visualize and analyze the clustering solutions. The former tool focuses on individual publications, while the latter concentrates on a collective scenario (Van Eck and Waltman, 2017). Finally, we also retrieved qualitative data of graduates’ opinions from a questionnaire.

### The methods *via* VOSviewer and CitNetExplorer

Researchers can establish the clusters and analyze the clustering solutions by combining CitNetExplorer (Van Eck and Waltman, 2014a,b) with VOSviewer (Van Eck and Waltman, 2010, 2014b). CitNetExplorer can categorize the publications using the clustering techniques based on direct citation networks, while VOSviewer can analyze and visualize the clustering solutions to rank authors, sources, documents, organizations, keywords, and countries based on either citation scores or link strengths. The types of analysis in VOSviewer include co-authorship, co-occurrence, citation, bibliographic coupling, and co-citation, while the counting methods include both full and fractional counting. CitNetExplorer mainly analyzes citation patterns in scientific literature using the documents sourcing from Web of Science. Its major functions include visualizing and drilling down into specific citation networks and searching for target documents except for clustering solution analyses.

VOSviewer and CitNetExplorer can be combined to conduct bibliometric analyses (Van Eck and Waltman, 2017). To conduct a bibliometric analysis using VOSviewer at a collective level, we searched the Core Collection of Web of Science and obtained 880 results on December 12, 2021. The search query included “artificial intelligence” OR AI (topic) and educat* OR teach* OR learn* (topic) and ethic* (topic) according to Boolean Operator rules. The type of documents included articles (*N* = 487), proceedings papers (*N* = 181), review articles (*N* = 168), editorial materials (*N* = 46), early access (*N* = 39), book chapters (*N* = 5), letters (*N* = 4), data papers (*N* = 2), book reviews (*N* = 1), and news items (*N* = 1). There might be overlapping since some studies were interdisciplinary. To conduct the specific analysis at an individual level, we removed the documents of “early access” to avoid the technical null pointer exception and finally included 841 results for the analysis through CitNetExplorer. Due to the inconsistencies in the findings of the literature, a comprehensive search and analysis are necessary for a bibliometric analysis (Keathley-Herring et al., 2016). Researchers, therefore, included all kinds of obtained documents including book chapters, editorial materials, and conference proceedings for a bibliometric analysis although they are not always peer-reviewed and rigorous as journal articles.

The results could be categorized into various research orientations. They included Computer Science Artificial Intelligence (*N* = 143, 16.140%), Computer Science Interdisciplinary Applications (*N* = 76, 8.578%), Computer Science Theory Methods (*N* = 73, 8.239%), Computer Science Information Systems (*N* = 56, 6.321%), Education Educational Research (*N* = 54, 6.095%), Ethics (*N* = 54, 6.095%), Engineering Electrical Electronic (*N* = 51, 5.756%), Health Care Sciences Services (*N* = 42, 4.740%), Radiology Nuclear Medicine Medical Imaging (*N* = 40, 4.515%), Medical Informatics (*N* = 34, 3.837%), Education Scientific Disciplines (*N* = 32, 3.612%), Medicine General Internal (*N* = 32, 3.612%), Multidisciplinary Sciences (*N* = 31, 3.499%), Philosophy (*N* = 31, 3.499%), Law (*N* = 30, 3.386%), Social Sciences Biomedical (*N* = 26, 2.935%), Social Sciences Interdisciplinary (*N* = 26, 2.935%), and Management (*N* = 24, 2.709%), etc.

### The questionnaire

We randomly selected 33 graduates (2 doctoral students, 6.1% and 31 post-graduate students, 93.9%), ranging from 21 to 40 years old (*M* = 23.48, S.D. = 3.633). The number of the population of graduates is around 45. We believe the sample is representative of the population since the percentage is high (73.33%) and the selection is randomly conducted. After signing the consent form, they were requested to fill in the blanks by freely indicating their opinions on the ethics of the use of AI in education. They were requested to write down their opinions using more than 500 English words and complete it within 2 h.

After completing the blank filling, they were requested to submit it directly through The Questionnaire Star portal or directly email to the researcher in case any technical problem occurred. Questionnaire Star, developed by Changsha Ranxing Information Technology Co., Ltd., can be used to collect data through questionnaires, online exams, and question-answers. It supports mobile filling, WeChat group discussion, rewarding, and data analyses anonymously. It can automatically save and record the data and keep all the responses confidential. Finally, the researcher collected 33 questionnaires and 18,479 words. Two experienced raters evaluated the reliability and validity of the responses. They excluded the unreliable and invalid responses. The unreal responses were also excluded. Both raters reached a satisfactory level of inter-rater reliability (*k* = 0.91). If both of them failed to reach an agreement on any choice, a third rater would be invited to make a final decision. Most of them were considered valid and reliable since most graduates wrote more than 500 English words with diligence and integrity under the supervision of the researcher.

A six-step thematic analysis was adopted to analyze participants’ responses. Firstly, researchers got familiar with the data by reading through the texts and taking notes. Secondly, they coded the data by highlighting the important words, phrases, lines, or sections. Thirdly, they generated themes from the data by combing codes. Fourthly, they reviewed the summarized themes and made sure the themes were representations of participants’ opinions. Fifthly, they gave a concise and accurate name to each theme. Finally, they wrote up the analysis of the responses in the result section.

### The PRISMA framework

**Figure 1:**
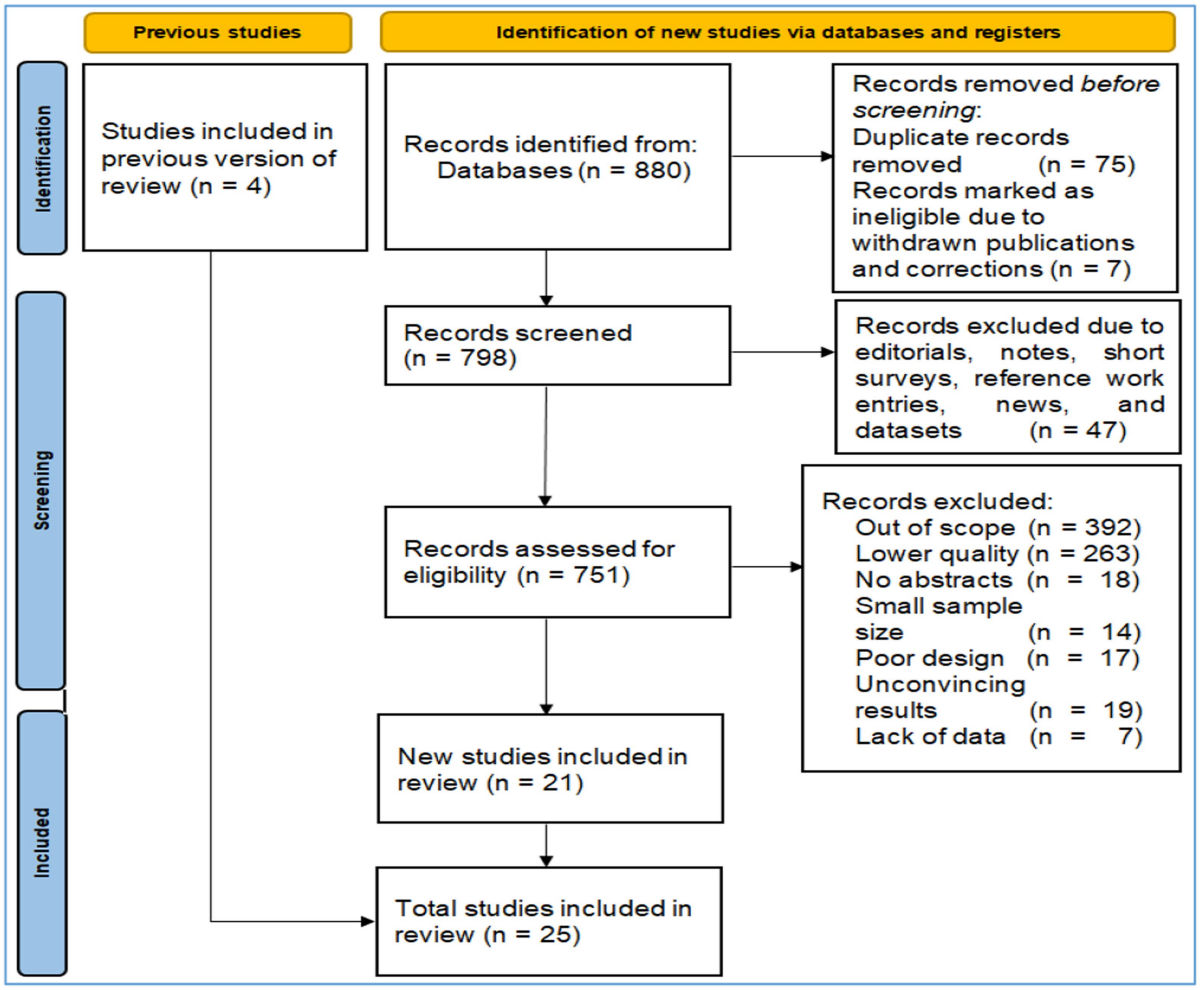
A flow chart of literature selection based on inclusion and exclusion criteria.

We adopted the PRISMA framework to include the studies. As shown in Figure 1, we firstly obtained 880 studies by keying in “artificial intelligence” OR AI (topic) and educat* OR teach* OR learn* OR pedagog* (topic) and ethic* (topic). Then we excluded those ineligible by Endnote (*n* = 13), removed the duplicates (*n* = 11), and deleted 9 results due to various reasons such as lower quality, irrelevance, and missing information. We excluded and included studies based on both inclusion and exclusion criteria. We included the studies if they (1) focused on the AI ethics in education, (2) could provide enough relevant information, or (3) were rigidly designed and arrived at convincing conclusions. We would exclude the studies if they (1) were duplicate records, (2) were records marked as ineligible due to withdrawn publications and corrections, (3) were classified as editorials, notes, short surveys, reference work entries, news, and datasets, (4) were out of scope, of lower quality, without abstracts, of a small sample size, poorly designed, with unconvincing results, or without enough data. Two raters evaluated and selected the literature and their decisions reached a satisfactory inter-rater reliability (*k* = 0.84). If both of them could not reach an agreement on any decision, a third rater would be invited to make a final decision. Finally, they included 25 results for the systematic review (Table 1).

**Table 1:** Details of the included studies.

*N*	Study	Source	Title
1	Burton et al. (2017)	AI Magazine	Ethical considerations in artificial intelligence courses
2	Asimov (1950)	Gnome Press	I, Robot
3	Mittelstadt et al. (2016)	Big Data & Society	The ethics of algorithms: mapping the debate
4	Floridi et al. (2018)	Minds and Machines	AI4People—An ethical framework for a good AI society: opportunities, risks, principles, and recommendations
5	Jobin et al. (2019)	Nature Machine Intelligence	The global landscape of AI ethics guidelines
6	Frankena (1963)	Prentice-Hall	Ethics
7	Aristotle (1999)	Hackett Publishing	Nichomachean ethics
8	Annas (2006)	Oxford University Press	Virtue ethics
9	Anscombe (2005)	Academic Press	Modern moral philosophy
10	MacIntyre (2007)	University of Notre Dame Press	After virtue: a study in moral theory
11	Turilli and Floridi (2009)	Ethics and Information Technology	The ethics of information transparency
12	Tutt (2016)	SSRN Scholarly Paper, Rochester, NY: Social Science Research Network	An FDA for algorithms
13	Tene and Polonetsky (2013)	Northwestern Journal of Technology and Intellectual Property	Big Data for all: privacy and user control in the age of analytics
14	Schermer (2011)	Computer Law & Security Review	The limits of privacy in automated profiling and data mining
15	Van Wel and Royakkers (2004)	Ethics and Information Technology	Ethical issues in web data mining
16	Riyal (2019)	Journal of Politics and Law	A brief analysis of the essence of education and human ethics-Hegel’s view
17	Holmes et al. (2021)	International Journal of Artificial Intelligence in Education	Ethics of AI in education: toward a community-wide framework
18	Pelczynski (1984)	Cambridge: CUP	Introduction: the significance of Hegel’s separation of the state and civil society
19	Siljander et al. (2017)	Rotterdam, The Netherlands: Sense Publishers	Schools in transition: linking past, present, and future in educational practice
20	McGowan (1991)	Ithaca: Cornell University Press	Postmodernism and its critics
21	Berry (2003)	Washington, DC: American Psychological Association	Conceptual approaches to acculturation
22	Berry and Sam (1997)	Needham Heights, MA: Allyn & Bacon	Acculturation and adaptation
23	Handelsman et al. (2005)	Professional Psychology: Research and Practice	Training ethical psychologists: an acculturation model
24	John-Mathews (2022)	Technological Forecasting and Social Change	Some critical and ethical perspectives on the empirical turn of AI interpretability
25	Følstad et al. (2021)	Computing	Future directions for chatbot research: an interdisciplinary research agenda

## Results

### RQ1. What is the trend of publications of ethics in the use of AI in education?

On December 27, 2022, researchers obtained 724 results from Web of Science by entering “artificial intelligence” OR AI (topic) and educat* (topic) and ethic* (topic) into the search column according to the Boolean Operator rules, ranging from 2007 to 2023. The types of results included Article (*n* = 564), Meeting (*n* = 127), Review Article (*n* = 84), Other (*n* = 32), Early Access (*n* = 32), Editorial Material (*n* = 20), Unspecified (*n* = 4), Book (*n* = 2), Case Report (*n* = 1), Letter (*n* = 2), Clinical Trial (*n* = 2), Correction (*n* = 1), Data Paper (*n* = 1), and News (*n* = 1).

**Figure 2:**
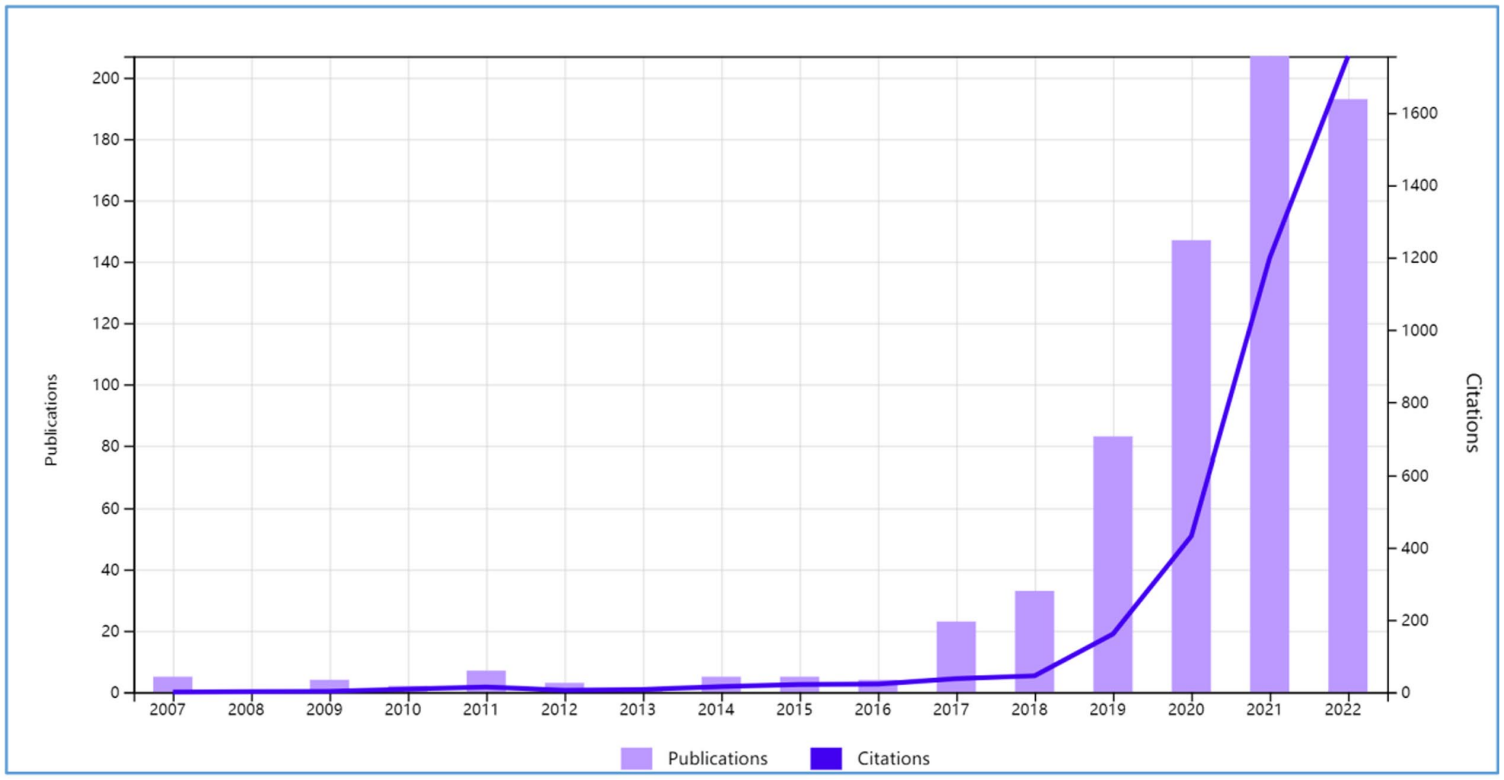
The trend of publications regarding ethics of AI for educational purposes.

Publications related to ethics of the use of AI in education have been gradually increasing since they emerged in the year 2007. The number of relevant studies slowly went up until 2017 when researchers suddenly authored far more publications than before. After 2017, the number of relevant studies rocketed up until the peak in the year 2021. It is noteworthy that the number of publications in 2019 nearly doubled that in 2018 possibly due to the drastic development of AI technologies. Citations nearly kept pace with publications. The period between 2018 and 2021 also witnessed a dramatic increase in the number of citations (Figure 2).

### RQ2. What are the top authors, sources, organizations, and countries?

**Figure 3:**
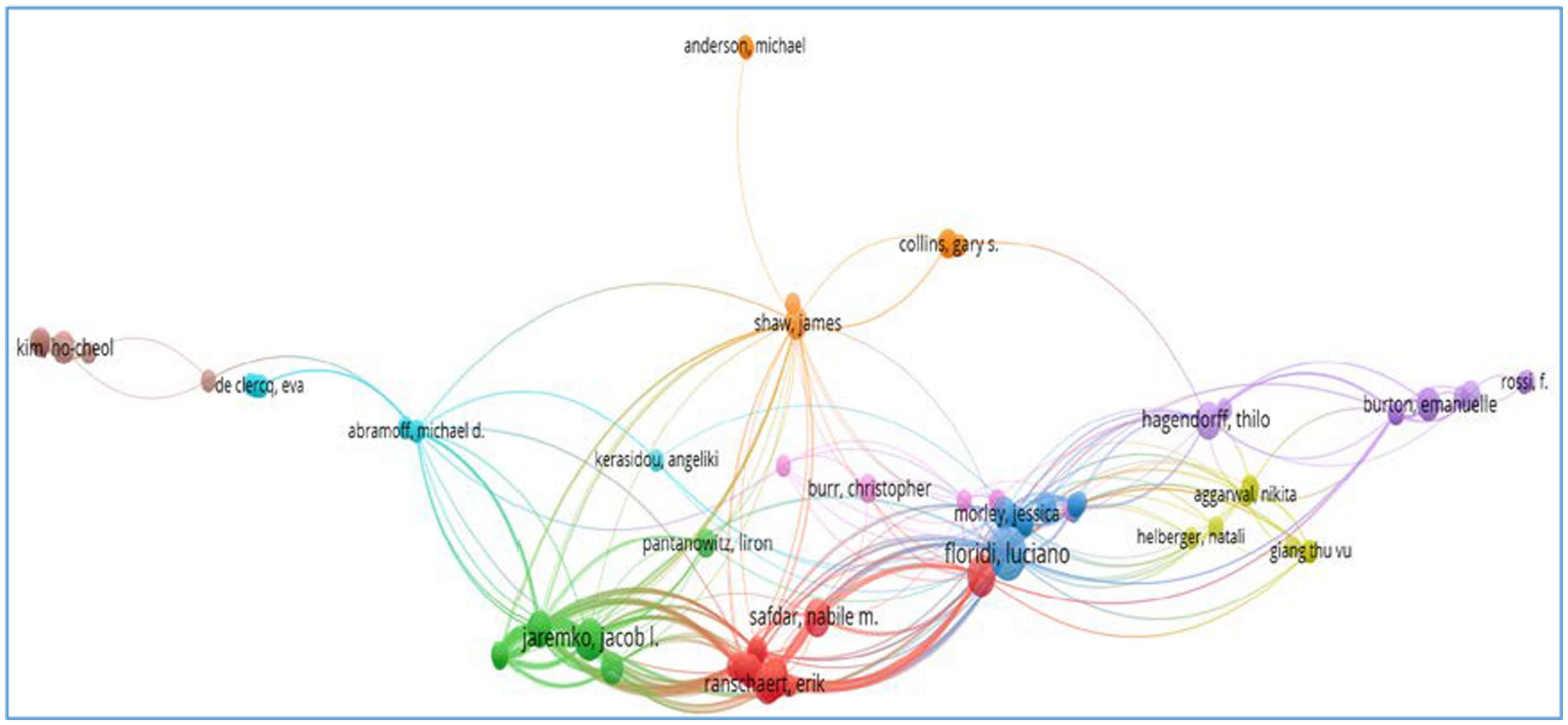
The citation networks of authors.

**Figure 4:**
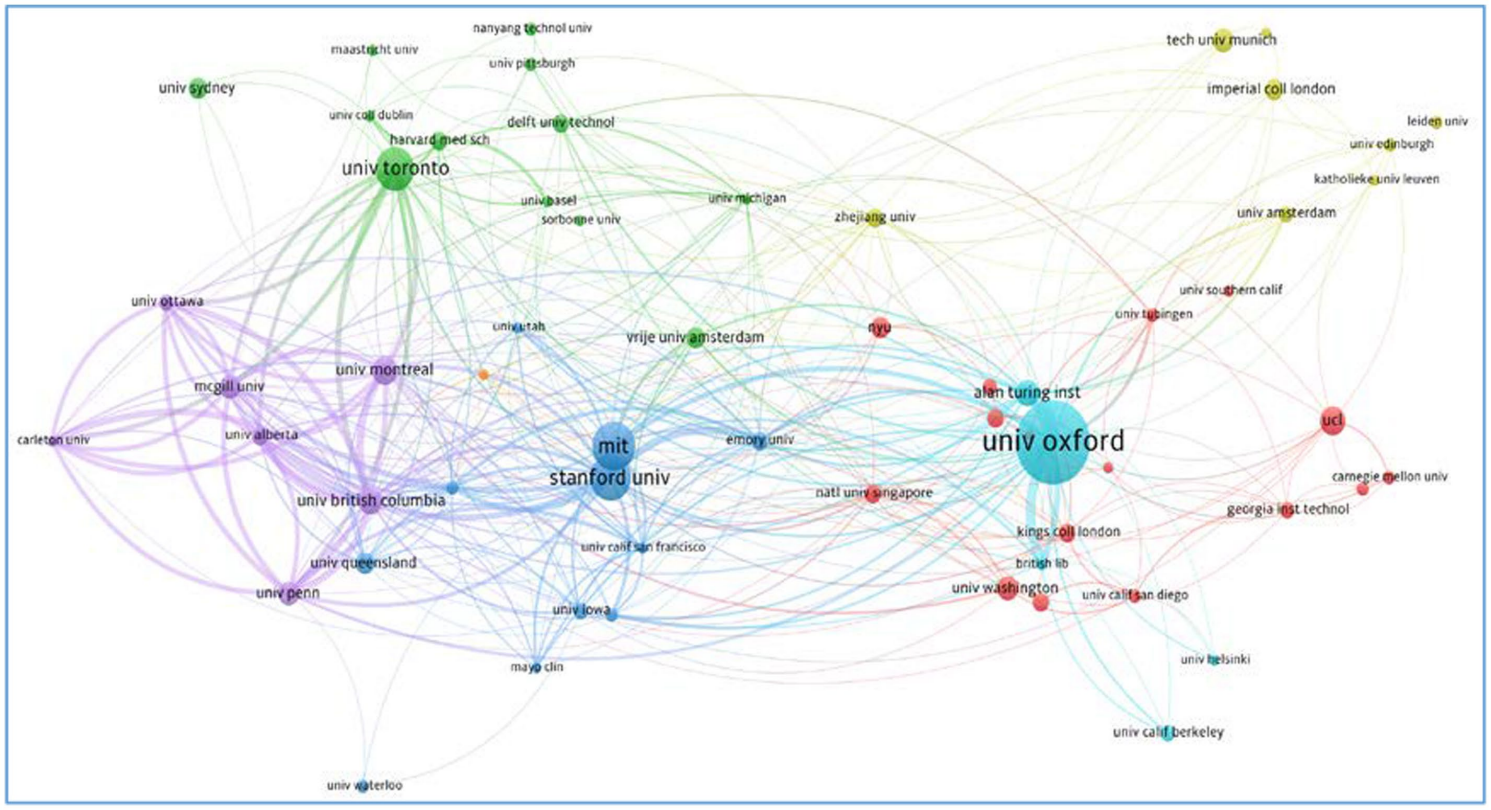
The citation networks of organizations.

**Figure 5:**
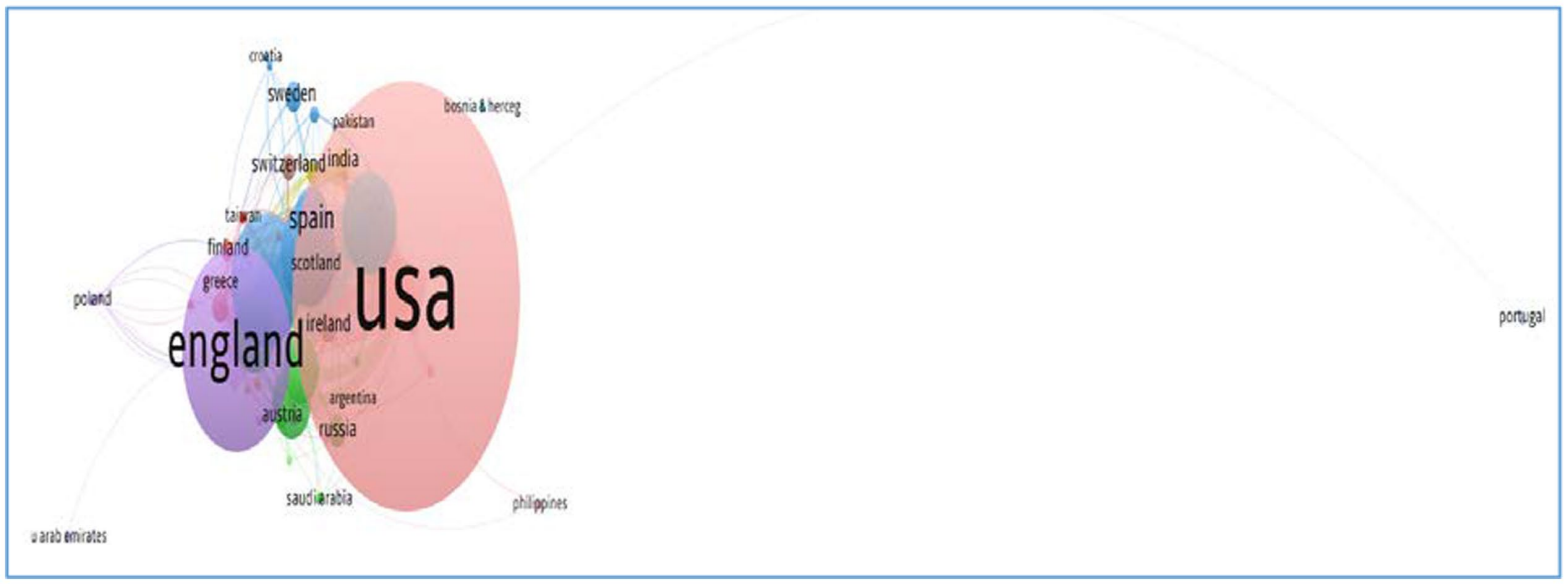
The citation networks of countries.

We used VOSviewer to analyze the clustering solutions in terms of authors, organizations, countries, and cited sources at an aggravate level. We selected co-authorship as the type of analysis, and authors, organizations, and countries as units of analysis. Then we obtained the top 10 authors (Figure 3), organizations (Figure 4), and countries (Figure 5) based on the citations. We also obtained the top 10 cited sources by selecting co-citation as the analysis type and cited sources as the analysis unit (Table 2). This could provide references for researchers, who could pay special attention to the top-ranking authors, organizations, countries, and cited sources when they attempted to conduct their future research. Another bibliometric analysis tool, i.e., CitNetExplorer, was adopted to cluster the publications and establish citation networks.

**Table 2:** Top 10 authors, sources, organizations, and countries.

*N*	Author	Cit.	Link	Cited source	Cit.	Link
1	Floridi, Luciano	280	10	Nature	474	3,339
2	Taddeo, Mariarosaria	199	8	Journal of the American Medical Association	361	2,985
3	Geis, J. Raymond	190	19	Science	349	2,177
4	Kohli, Marc	190	19	Plos One	297	2,584
5	Safdar, Nabile M.	99	18	Artificial Intelligence	257	925
6	Jaremko, Jacob L.	92	24	Nature Medicine	252	2,277
7	Tang, An	92	24	Lecture notes in computer science	230	1,072
8	Morley, Jessica	90	6	Journal of Medical Internet Research	222	1858
9	Hagendorff, Thilo	90	0	New England Journal of Medicine	220	1942
10	Kim, Ho-cheol	85	0	NPJ Digital Medicine	170	1869
*N*	Organization	Cit.	Link	Country	Cit.	Link
1	University of Oxford	543	12	The USA	2,728	168
2	University of Montreal	407	14	England	1,534	132
3	The Alan Turing Institute	342	11	Germany	853	100
4	Stanford University	227	6	Canada	750	73
5	Massachusetts Institute of Technology	181	8	The Netherlands	559	89
6	University of Toronto	153	15	Italy	541	60
7	University College London	126	7	France	527	56
8	University of Pennsylvania	96	15	Australia	498	64
9	University of Washington	84	1	Spain	348	37
10	Technology University Munich	74	0	China	166	25

Researchers explored the citations networks of authors (Figure 3). Authors who form large citation networks are Floridi Luciano, Ranshaert Erik, Jaremko Jacob I, Morley Jessica, Hagendorff Thilo, Pantanowitz Liron, Shaw James, and Collins Gary S. Anderson Michael, Kim Ho-Cheol, De Clercq Eva, Rossi F, and Burton Emanuelle are distant from the major citation networks although they are associated with others. It is noteworthy to indicate that Anderson Michael is relatively less associated with major citation networks although this author is connected to that headed by Shaw James. Some authors, e.g., Rossi F, and Burton Emanuelle, are closely related although they remain far away from the major citation networks.

Researchers studied the citation networks of organizations that authors were affiliated to (Figure 4). University of Oxford has the largest citation networks, followed by Stanford University, Massachusetts of Institute, and University of Toronto. There are also relatively larger citation networks centered by University of British Columbia, University of Queensland, University of Pennsylvania, Harvard Medical School, University of Sydney, University of Pittsburgh, Nanyang Technological University, Imperial College London, and University of Pittsburgh. Leiden University, University of Edinburgh, University of California, Berkeley, and University of Waterloo remain far away from the center of citation networks and are weakly connected to major citation networks.

Researchers analyzed the networks of countries where artificial intelligence ethics underwent explorations in education (Figure 5). Ethics of artificial intelligence experienced extensive examinations in many countries and areas. The network of citations demonstrates that there are limited relationships between countries and areas. Authors from the USA present more publications than those from other countries, followed by those from England and Spain. There are close citations among authors from the USA, Ireland, India, Argentina, Russia, Scotland, Spain, India, and Pakistan. Authors from Portugal are isolated in terms of citations. Authors from United Arab Emirates, Saudi Arabia, the Philippines, Croatia, and Poland are less associated with the major publications. Another major citation network centers on England, connected to Greece, Finland, and Australia.

### RQ3. What is the essence of ethics of AI used in education?

**Figure 6:**
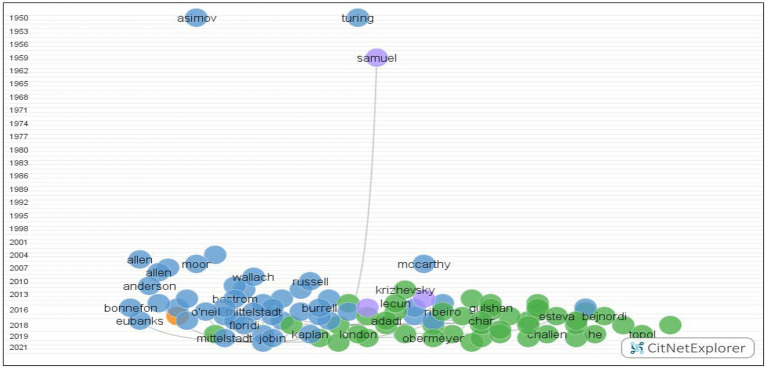
A clustering map based on citation networks.

Four clusters were established using the clustering techniques in CitNetExplorer. The obtained data were firstly entered into CitNetExplorer. The results included non-matching cited references and the minimum number of citations was set at 10. Then the citation networks were established after which researchers obtained the clusters through the clustering technique in CitNetExplorer. The clustering techniques categorized the publications into four groups ranging from 1950 to 2021, and 273 publications did not belong to any cluster due to the requirement on the minimum size (Figure 6). We included the publications totaling 944 with 2,149 citation links. Group 1 included 320 publications, 789 citation links, and 23 publications with higher than 15 citation scores; Group 2 included 310 publications, 1,001 citation links, and 21 publications with higher than 15 citation scores; Group 3 included 28 publications, 27 citation links, and 2 publications with higher than 15 citation scores; Group 4 included 13 publications, 15 citation links, and no publications with higher than 15 citation scores. Considering the large number of publications and citation links, we, therefore, focused our analyses on the first two groups, leading to summarized themes, i.e., the essence and principles of ethics of AI used in education.

The essence of ethics of AI used in education should be determined before ethics principles were formulated. The essence of ethics is descriptive, whose research methods can be classified into three dimensions, e.g., deontological, utilitarianism, and virtue perspectives. Rather than independent or exclusive, the three dimensions are mutually correlated. Ethical education should attempt to comprise all of the three dimensions. Teachers should try to cultivate students in multiple aspects in terms of ethical judgments. When judging a particular issue, they should inclusively consider the ethical norm and aim for the most suitable solution (Burton et al., 2017) based on the essence of ethics.

### Deontology

As for deontology, ethics is perceived as a norm based on the responsibilities prescribed in a moral law originating from ancient divine codes of religions, e.g., Christianity, Judaism, and Islam (Ye et al., 2021). This deontology requires human beings to keep consistent with the prescriptions based on the code of ethics since the responsibilities are compulsory. Human beings could design ethical AI based on deontology (Asimov, 1950). Social rules could be integrated into ethical codes, paving a foundation for ethical norms used in the design of AI. AI could aim to maintain social orders and cultivate a harmonious environment for human beings and machines to interact with each other.

### Utilitarianism

Utilitarianism sheds light on the benefits of ethics for the individuals and society. It aims to strike an optimal balance between good and evil by formulating the ethical codes (Frankena, 1963). The assumption for this ethics is that utilities could be quantified into several sections, where individuals could adopt some of them to measure social benefits or levels of happiness. Any individual preferred to maximize their benefits by conforming to the related ethic codes. The utilitarianism individual could compare his or her own benefits with the overall social gain before a specific ethic choice was made. In this way, the utilitarianism ethics could facilitate the progress of society and individuals.

### Virtue ethics

Aristotle (1999) defined virtue ethics as the code with a concentration on objectives. Individuals developed ethical habits or made corresponding choices to achieve their goals. They tended to make the ethical choices that could make them work or learn smoothly or live happily (Annas, 2006). Different from deontological ethics, virtue ethics, highlighting local codes instead of universal standards, deemed something good from the perspective of individuals rather than the society. Virtue ethics focused on the quality of individual living and considered it an ability to foster individual life through practical experiences based on education or other skills. Virtue ethics has dominated the western world among well-educated people for centuries, further developed and advanced by Anscombe (2005) and MacIntyre (2007).

### RQ4. What are the principles of ethics in the use of AI in education?

We analyzed Group 1 based on five publications with top citation scores (Turilli and Floridi, 2009; Tene and Polonetsky, 2013; Mittelstadt et al., 2016; Tutt, 2016; Jobin et al., 2019). The international research community agreed upon five ethical principles in the use of AI for educational purposes, i.e., transparency, justice, fairness, equity, non-maleficence, responsibility, and privacy. These guidelines provided constructive directions for the use of AI in education, based on which designers and educators could explore and examine the use of AI for educational purposes. Different even controversial interpretations regarding the ethical principles have been proposed with the development of AI technologies since they were born (Jobin et al., 2019). However, there are generally agreed perceptions regarding the essence of each ethic guideline, as well as the role that each of them plays in the use of AI in education.

### Transparency

Transparency was defined by Turilli and Floridi (2009, p. 106) as the availability of information, the degree of availability, or the role in decision-making process. Transparency has become an eye-catching concern in the use of AI for educational purposes. Transparency is in general supported by most users and researchers since AI technologies are hard for laymen to control and perceive (Tutt, 2016). The initial elements of transparency included the understandability and availability of digital information (Mittelstadt et al., 2016). The information output should be understood by users and users should have easy access to the necessary information. However, the complete transparency may be difficult to be achieved since information always exists in the form of asymmetry and imbalance (Tene and Polonetsky, 2013, p. 252).

Of the five ethical principles, transparency is the most prominent one that guides the development of the use of AI in education. Transparency refers to the requirement that when AI is used in education or information transfer, the specific parameters, source, responsibility of the use of AI, investment, and impact of AI should be disclosed to enhance accountability, modification, interpretation, and communication between AI users, educational researchers, and practitioners (Jobin et al., 2019). Under the guideline of the transparency principle, users expect AI technologies to disclose any potential, possible, or real disadvantages or risks in the use of AI in education. Educators have the right to make a decision on whether to use them or not. Transparency requires the information available and open to users, which is contrary to privacy.

### Privacy

Privacy is considered an important factor that needs to be valued. Privacy is closely related to the free access to personal information and the ability to keep it under control. Designers of AI and AI-assisted educators could value the necessity to protect privacies of students or learners. Their personal information, e.g., mobile number, gender, birthday, and age, should be kept secret to avoid personal harassment. Privacy is closely related to data protection and security. AI designers should try to protect data and ensure personal security. Disclosure of private data may cause harms to users of AI. Designers and educators could also try to enhance the privacy awareness and data regulations (Jobin et al., 2019). With solid privacy protection mechanisms, users of AI could benefit from the development of high technologies.

With the fast development of AI technologies, privacy has growingly attracted researchers’ attention. The movement against discrimination, equal sharing of educational resources and transparent negotiation requires the privacy of personal information (Schermer, 2011). Personal information privacy depends on the ability of users to refrain themselves from revealing their personal information (Van Wel and Royakkers, 2004), and the difficulty in disclosing their personal information to others. The advanced data analytics could reveal personal identities based on the accumulated data regarding the analysis of behaviors. It is, however, difficult to hinder the rapid development of techniques of analytics of data. Personal privacy has, therefore, become a serious concern in the use of AI in education.

### Justice, fairness, and equity

Justice in the use of AI in education is closely connected with the distribution of educational equipment, learning resources, and faculty members. The AI-based educational equipment should be arranged in an unbiased manner, bringing benefits to all levels of students and teachers. The learning resources could be delivered to everybody through the AI technology-supported online platforms or other methods (Yu, 2022). Faculty members who have higher academic levels should be distributed to different levels of students, who might then have easy access to AI-assisted delivery of knowledge. Montreal Declaration highlights the importance of justice by stating, “The development of AI should promote justice and seek to eliminate all types of discrimination,” and Asilomar Principles promote shared benefits and developments based on AI technologies. It was argued that AI technologies should improve international justice and equal benefits (Floridi et al., 2018).

Justice could be improved through various methods. Examples are technical standards or coding, public awareness of security and regulation, data protection, laws or rules, governmental regulation, interdisciplinary cooperation, and interactive efforts (Jobin et al., 2019). Justice also indicates fairness and equity in the educational use of AI. Users should strike a balance with others in terms of benefits, priorities, or gains. The access to AI use should be open to everybody who may decide whether to adopt AI in his or her education or not. Potential users should also be informed of the potential harms, disadvantages, or any possible negative effect in the use of AI for educational purposes.

### Non-maleficence

AI used in education is expected to be in line with the non-maleficence. The ethical guideline of non-maleficence requires that AI should not bring any harm to human beings in various fields such as education. Users should also adopt risk or harm management strategies to avoid using AI that may cause any potential harms and make human beings run any risk. Harm is of many varieties such as discrimination, physical or private violations, distrust, non-skillfulness, or any negative effect on infrastructure, regulation, social welfare, psychological state, emotion, or economy (Jobin et al., 2019). This ethical guideline proposes a highly demanding requirement for designers and users to manufacture or select the AI technologies for educational purposes.

To distinguish the conception of non-maleficence, we should firstly identify the two seemingly identical statements “do only good” and “do no harm.” The former logically indicates beneficence, while the latter logically indicates non-maleficence. Beneficence requires that AI merely contribute goodness to human beings and society by sharing benefits and welfare among social members. Non-maleficence requires that the use of AI can neither cause anything harmful to human beings and society nor hinder the advancement of society and technologies. A special concern is the protection of personal data privacy, which is easily violated in the use of AI technologies once AI is beyond a strict control (Floridi et al., 2018).

### Responsibility

Responsibility is considered essential in the use of AI in education. Developers could assume the responsibility to make people share the benefits, advantages, usefulness, and anything good of AI technologies. The lack of relevant responsibility would bring about unexpected risks or harms, especially when the AI system operation is beyond human perceptions and controls. The IEEE file encourages designers and educators of AI to “avoid misuse” of AI technologies, and the Montreal Declaration states that the developers and designers of AI technologies “should assume their responsibility by working against the risks arising from their technological innovations,” which has been echoed by the academic community (Floridi et al., 2018). Specific items of responsibility might be clearly described before experts determined to develop AI.

Responsibility should be taken into serious consideration of developers and designers. While very few studies defined responsibility, it is generally deemed as integrity and legal duty that may be prescribed in a contract. This ethical guideline suggests users of AI attempt to assume social, interpersonal, or academic responsibilities and minimize the underlying factors that may cause harms to individuals or communities. Users can also highlight diversified needs and introduce the ethical responsibility into various educational fields. Educators and developers of AI should shoulder the responsibility for any harm caused by the use of AI in education (Jobin et al., 2019). They should make every effort to create human-like AI assistants that may bring about more benefits than harms to education.

## Discussion

### Research trends and ranks in AI ethics in education

AI ethics in education is developing rapidly, facilitated by the rising trend of AI technologies. Ethics as a subject is also forming a dramatic catalyst to capture researchers’ attention and interest. AI ethics in education has been closely related to cognitive sciences, machine learning, and teaching paradigms across North America, Asia, and Europe (Javed et al., 2022). To promote the healthy development of AI, educators can make every effort to enhance students’ awareness of the importance of AI ethics by integrating AI ethics courses into the educational system. Developers can then design and produce AI technologies according to ethics principles and quintessence to provide better AI services for education.

The responses from the questionnaires are in general consistent with the bibliometric analysis results. Participants expressed that AI ethics had caught attention of a growing number of researchers and showed their concerns with AI ethics in education. A participant wrote “Considering the irresistible trend of applying artificial intelligence to pedagogy, we may think about some questions: will artificial intelligence replace teachers? Does it only exert a positive effect on learning outcomes? The answer is definitely not, because every coin has two sides, and artificial intelligence is not an exception.” Participants were also aware that the popularity of AI use could solve the problem of the imbalanced allocation of educational resources. For instance, a participant wrote “We know that insufficient allocation of educational resources in our country is a problem to solve. In some schools, one teacher might have to deal with the studies of dozens of students. While with the help of AI, before the one-to-one tutoring between teachers and students, AI can alleviate the problem by virtue of solving common problems of students.”

No participants expressed their opinions on the rankings of authors, sources, countries, and organizations in AI ethics in education. This indicates that they are not concerned with the academic frontiers of AI ethics in education since most of them are interested in pedagogical approaches integrated with AI ethics. Lecturers tended to deliver research methods and topics such as AI ethics in education, linguistics, phonetics, and phonology rather than the frontier knowledge regarding AI ethics in education. Students received the input passively rather than actively sought cutting-edge insights. It may be a wise decision to cultivate students’ insights into frontier knowledge and encourage them to explore the knowledge actively and comprehensively.

### The essence of ethics of AI used in education

Ethics and education are mutually connected in the development of society. On one hand, ethics could improve the quality of education and higher quality of education could in turn enhance the ethics. On the other hand, the co-existence and development of ethics and education could improve the integrity of social development, where AI development could be healthily promoted toward the balance between ethics and education. Ethics, in essence, is the clearance of individualism and selfishness to merge individuals with social development, establishing the ethical entity cultivated during the long-term human development and historical evolution (Riyal, 2019). Education is an important channel to achieve the ethical entity (Holmes et al., 2021), where human ethics could be realized with the development of human happiness and satisfaction in the evolution of AI technologies.

The essence of ethics in AI used in education is linked to three aspects, i.e., law and moral, civil society, and the state. The regulations of law establish the lower bound of ethical requirements. Anybody violating the law will also be considered having violated ethics. Morals, however, are the upper bound of ethics. People who violate the morals do not necessarily infringe on ethics. AI ethics in education should fluctuate between law and moral. AI ethics could be the integration of abstract law and morals (Pelczynski, 1984, p. 201–203). Ethics, linking to civil society, is characterized by social interactions, where the ethical codes gradually emerge.

The ethics of the state is the top-most level of AI ethics for educational purposes. The link of ethics to the state is built upon the connection between moral, law, and society. The latter interactions finally lead to the ethical entity of the state, where individuals may live and society may operate normally. An individual rich life in the society provided a baseline for humans to form social ethics, developing into state-based ethics, which in turn ensured the continuance of individual and social ethics (Siljander et al., 2017, p. 98). The individual and social ethics should thus aim at the ethics of the state, the universal objective, which could then sustain individual rights and social equity (McGowan, 1991, p. 59).

### The principles of ethics of AI used in education

The cultural adaptation model and acculturation (Berry and Sam, 1997; Berry, 2003) could be combined to form an acculturation model of ethics of AI in education (Handelsman et al., 2005). The acculturation model included two main constructs, i.e., maintenance and participation. The former indicates that educators or learners maintain their traditional ethical values based on their cultural traits such as race, familial backgrounds, nationality, religions, and social class. The latter indicates that educators or learners should acknowledge and adopt their traditional cultural values and follow the cultural values as the ethical principles in the use of AI in education (Handelsman et al., 2005).

Learners and educators tend to adopt an integrated model in case they remain at a higher level of maintenance and participation. Maintenance and participation are closely related to each other during the learning process. When learners and educators tend to maintain a higher level of ethics of AI in education, they may consciously or unconsciously borrow elements or traits from their embedded cultures. Cultures, deeply rooted in their awareness and habits, may exert a great influence on their maintenance of ethics. Educators and learners will form in-depth perceptions toward interactive cultural traits and gradually develop toward the acculturation model (Handelsman et al., 2005, p. 60). In this way, the acculturation model of ethics of AI used in education will be cultivated and guide the development of ethical AI use for educational purposes (Berry and Sam, 1997).

### Balance of ethical essence and principles

It is important to strike a balance between various elements of the essence and principles of AI ethics in education. A participant wrote, “Considering artificial intelligence used in education, some of its limitations should be seriously weighed. As for how to weigh the essence of artificial intelligence used in education and principles of ethics, I think the principles of ethics are the baseline of artificial intelligence, and the transparency, privacy, and justice cannot always be ensured when utilitarianism of artificial intelligence is regarded as the primary function.” The essence of AI ethics should be inhibited if ethical principles lead the developmental orientation of AI used in education.

Besides, an algorithm is an important factor to be included in the use of AI in education. Another participant responded, “The algorithm interference suggests it is difficult to avoid injustice, unfairness, and inequity. In the intelligent era, more attention should be paid to algorithms. Only in this way can artificial intelligence be better served for education.” When applying AI to education, what should be noticed is that human is the focus and more attention should be paid to human behaviors and thoughts rather than simply the deterioration of humans as a tool of machine. Designers should attempt to develop AI toward the modification of human behaviors and thoughts based on humane algorithms. In view of the utility of AI, algorithms consistent with the ethical principles such as transparency, privacy, justice, fairness, and equity should also be taken into consideration. Without the interference of human algorithms, the unethical problems of AI used in education might be unavoidable.

### Interactions of the elements in the essence of AI

Utilitarianism could pave a solid foundation for close interactions between other essential elements in AI ethics in education. From the perspective of human orientation, the essence of AI, to a large extent, lies in its utilitarianism, especially when the economy prospers. A participant wrote, “My highlight on the practical aspect of AI does not mean that I deny the essential fact that AI is also making a difference in other perspectives, say, deontology and virtue, which, in nature, are in dialectical complementation with utilitarianism.” Users could make convenient use of AI technologies in education. Students do not bother to write with pens and paper, which may spare the teacher the trouble of identifying the fuzzy handwriting. When increasingly more learners are taking advantage of AI, the attention to deontology and virtue is necessary since they can play an important role in the interaction with utilitarianism of AI and cultivate a benign picture of the use of AI in education.

### Principles of AI ethics in education

It is noteworthy that some participants argued that privacy might be the first important principle guiding the ethics of AI in education. A participant wrote, “Nevertheless, in the course of enjoying the enormous benefits AI provided, people diverged greatly in viewing the principle of ethics including information transparency, user’s privacy, justice, fairness, equity, non-maleficence, and responsibility. As I see, the advantages of AI-aided education overweigh its disadvantages.” Proper regulation may keep privacy under control in case the information is not evenly distributed among educators. The information boom requires educators and learners to possess the capacity of accessing learning resources and being aware of the potential revelation of personal privacy. Self-defense of privacy is also considered an important skill in the information-overwhelming age.

As education is becoming increasingly accessible with the aid of AI, the principles of education such as information transparency, justice, equity, and fairness are gradually emerging in that AI technology can reveal many issues previously hidden. This indicates that information should keep available to everybody who needs it, where obtainers indiscriminately share information regardless of the social class, economic status, or educational background. However, it seems hard to achieve absolute justice, equity, and fairness in information access. Learners in poverty-stricken areas may be not equipped with Internet connection devices. The transparency of information may not make any sense in such a situation. Educators should make every effort to promote equity and fairness under the principle of information transparency. When controversies arise, justice should be maintained in the use of AI in education.

In addition, non-maleficence and responsibility are best mirrored in the AI technology-assisted online learning and teaching. A participant wrote, “In the right path of AI development, every ‘traveler’ bears its responsibility, heavy or light, to make the education circle a better place; a small deed such as not intruding others privacy and making every attempt to invent a software, will make all the difference.” The development of AI should not do harm to human beings. Developers and educators should thus assume the responsibility that AI technologies keep under control and benefit human beings wherever AI technologies march, whatever AI they develop, and whomever they educate with AI technologies. Principles of non-maleficence and responsibility coexist and play an important role in the use of AI in education.

### Future research agenda

Future research can consider the influence of AI interpretability on AI ethics in education because the ability to interpret the AI decisions can help judge whether the decision is consistent with ethical criteria (John-Mathews, 2022). Future research can include more elements of AI ethics in education, e.g., non-discrimination (Hagendorff, 2020) and accountability (Ntoutsi et al., 2020). Future research can also try to address these questions (Følstad et al., 2021): (1) what are the potential ethical issues if AI technologies imitate human behaviors and thoughts? (2) What values can be included when designers develop global AI tools or advanced robots? (3) What can be considered when AI technologies replace humans for educational purposes? (4) What can be considered when AI is used in education for decision making?

Future research can highlight the development of mechanisms to measure the trustworthiness and acceptance of the use of AI in education. Some educators and learners still refuse to accept the use of AI for educational purposes. Designers and teachers can measure the acceptance and trustworthiness of AI used for educational purposes to enhance the popularity and acceptance of AI in education. Theoretical models, acceptance models, and trustworthiness models can be developed to measure whether AI can successfully be applied to education. Learners and teachers can apply AI to educational practice and theories based on solid support and benefits of AI can then be yielded (Kaur et al., 2023).

Future AI ethics governance can be developed based on enhanced AI ethics principles. With the rapid development of AI technologies, educators and learners may be confronted with continuously rising ethical issues. They may feel unfamiliar with the new ethical problems and find it hard to cope with all the potential ethical issues. It becomes important for them to govern the AI ethics according to escalating AI ethics principles. However, the principles may not be solid enough to deal with developing AI technologies. Information robustness, back-up governance measures, and adaptive governance policies may be necessary to govern the complicated AI ethical problems in education (Agbese et al., 2023).

## Conclusion

In this conclusion section, researchers will answer the research questions of the paper briefly and highlight the practical and theoretical implications of the paper, the contributions, and the limitations.

### Major findings

This study examined the essence and principles of AI ethics used in education, as well as the bibliometric analysis of AI ethics for educational purposes. The use of VOSviewer led the author to reveal the top 10 authors, sources, organizations, and countries in the research of AI ethics in education. The analysis of clustering solution through CitNetExplorer concluded that the essence of AI ethics for educational purposes included deontology, utilitarianism, and virtue, while the principles of AI ethics in education included transparency, justice, fairness, equity, non-maleficence, responsibility, and privacy. This provides a solid foundation for theoretical researchers to conceptualize the mechanisms to cope with AI ethical problems in education and for practitioners to find out ways to deal with rising ethical problems in the use of AI for educational purposes.

### Limitations

The most important limitation lies in the fact that this study could not include all the relevant documents due to the limited library resources. Resources of this study could not be extended to other databases such as Elsevier, EBSCO, and Engineering Village. This study also mainly included the studies written in English other than other languages.

## Data availability statement

The original contributions presented in the study are included in the article/Supplementary material, further inquiries can be directed to the corresponding author.

## Ethics statement

Ethical review and approval was not required for the study on human participants in accordance with the local legislation and institutional requirements. Written informed consent from the patients/participants or patients/participants legal guardian/next of kin was not required to participate in this study in accordance with the national legislation and the institutional requirements.

## Author contributions

LHY: writing, editing, and analysis. ZGY: conceptualization, design, and proofreading. All authors contributed to the article and approved the submitted version.

## Funding

This work was supported by 2019 MOOC of Beijing Language and Culture University (MOOC201902) (Important) “Introduction to Linguistics”; “Introduction to Linguistics” of online and offline mixed courses in Beijing Language and Culture University in 2020; Special fund of Beijing Co-construction Project-Research and reform of the “Undergraduate Teaching Reform and Innovation Project” of Beijing higher education in 2020-innovative “multilingual +” excellent talent training system (202010032003); The research project of Graduate Students of Beijing Language and Culture University “Xi Jinping: The Governance of China” (SJTS202108).

## Conflict of interest

The authors declare that the research was conducted in the absence of any commercial or financial relationships that could be construed as a potential conflict of interest.

## Publisher’s note

All claims expressed in this article are solely those of the authors and do not necessarily represent those of their affiliated organizations, or those of the publisher, the editors and the reviewers. Any product that may be evaluated in this article, or claim that may be made by its manufacturer, is not guaranteed or endorsed by the publisher.
