# Supplementary material for: Qualitative and quantitative analyses of artificial intelligence ethics in education using VOSviewer and CitNetExplorer
Source: Front Psychol. 2023 Mar 9;14:1061778. doi: 10.3389/fpsyg.2023.1061778 (PMC10035335; doi:10.3389/fpsyg.2023.1061778)
Supplement: Supplementary file 1 [file Data_Sheet_1.ZIP › dataset/opinions submitted.docx]

**Opinions from the questionnaires (anonymous)**

Since technology has developed a lot in recent years, especially the artificial intelligence which boasts a rapid growth nowadays, educators also try to adopt new technology like AI in education. And up to now, there are many achievement about AI in education. Students can choose online classes or traditional offline classes and they can also listen to other school's high quality courses due to the application of resource sharing platform. Teachers also have more teaching methods with the help of artificial intelligence. However, despite the technological problems, some moral issus are coming to light. When we use technology in education, we regard it as a tool to help us learning. It seems like the practicability ranks first in the process which lead to the thinking of the essence of this assistant. Utilitarianism only cares about the effects of the action. Whether there are some practical changes are brought with the behavior. To some extent, it is quite reasonable to be pragmatism. It promotes learning because learners will have concrete progress after the study. But we can't do anything against morality and law. The same is true of deontology. We can't say that the motive is right without considering the consequences, and we can't ignore the method for the sake of substantial results. Nowadays, as the Internet is full of all kinds of electronic resources, people can easily share them for free. But this raises questions about intellectual property. People should not use immoral ways to acquire knowledge. We should combine pragmatism and deontology, using proper technological means under the constraints of virtue. The same goes to the principles of ethnics. Now everyone is in the big data environment, what we do online all has a track record. This transparency can easily violate people's privacy. The school uses technology to collect students' basic information, which may include students' age, gender, family situation, personality, daily hobbies and so on. Once the information is not well preserved after being used for legitimate reasons, it is easy to leak out. Some software launched the function of taking photos and searching questions, so that when some students encounter a question that they cannot solve, their first reaction is not to think independently but to look for the answer through the Internet. Children in big cities are blessed with more resources, which will inevitably lead to educational inequality between poor and wealthy areas. For our country's college entrance examination system, this is not a good tendency. So from this point of view, I think the education department should gradually take measures to eliminate the educational imbalance if AI is put into widely use.But technology also has great advantages in education for example gaming education. Many popular games contain a lot of violence, which can be a bad way to mislead an immature child. Game teaching uses the way that children are most easy to accept, teaching in

The essence and principles of ethics of artificial intelligence used in education are necessary and sufficient. These essence and principles of ethics used in education are necessary for the following reasons. Firstly, education relies on artificial intelligence could get great development, but artificial intelligence used in education can not go beyond moral ethics. Secondly, artificial intelligence is a double-edged sword, and the same is true when artificial intelligence is used for education. So some ethics are needed to constrain artificial intelligence. It?s precisely because of these that the essence and principles of ethics of artificial intelligence used in education are necessary. These essence and principles of ethics used in education are the minimum limits on human nature?Based on these essence and principles, people define a safe space for artificial intelligence in education. Only in this safe zone, people can try their best to develop artificial intelligence technology about education. Otherwise, the extreme development of artificial intelligence used in education is nothing but a sword which would return to hurt human being themselves. These essence and principles of ethics of artificial intelligence used in education is really achieve better development of education rather than for other utilitarian or even some purpose with person selfish desires. On the other hand, the essence and principles of ethics of artificial intelligence used in education are sufficient. They make sure the boundary of science and destruction. The essence of ethics of artificial intelligence used in education are Utilitarianism, Deontology and Virtue. These are the demands of development. Artificial intelligence technology applied in the field of education has intelligent, automated highly accurate data analysis and processing capabilities and activate learning capabilities to undertake some of the work that only teachers can do in the past. So it is effectively to solve a series of problems faced by education. The principles of ethics of artificial intelligence used in education are transparency, privacy, justice, fairness, equity, non-maleficence, responsibility. Which not only put demands on teachers and students but also artificial intelligence servers. Teachers and students will all interact directly with educational artificial intelligence providing input to the AI system and receiving information from the systems output. They make decisions based on the information output of educational artificial intelligence. AI also have a monitor who is an agency or a person. The main work of him is to review the effectiveness and potential risks of application of education AI. So these principles are the demands of all these participants who participate in the educational activities. Transparency for the public, privacy for the participants. Responsible for the AI project, non-maleficence to the participants. These essence and principles of artificial intel

In modern society, artificial intelligence is playing an increasingly prominent role, we are becoming more and more familiar with its appearance in daily educational activities. Actually, in my opinion, the application of AI in education shows the essence and principles of ethics, and will be promoted even more widely in the future.Firstly, I think the use of AI in education follows the essence, which includes Utilitarianism, Deontology and Virtue. For students, with easy and quick approaches of intelligence devices, they can learn the same content from different dimensions, visual, audio or both. They can interact with teacher and partners in this process, which will facilitate improved retention of knowledge and encourage in-depth research and study. On top of this, as different students are equipped with different interests,gifts and abilities, AI can also offer special study plans and contents for them, provide unique suggestions for them and give notice for review. It will save much time and energy for rest or deepening their studies. For teachers, AI can be used to collect information from students for their preparation before class, enrich students’ experience in class and set aside homework and give guidance after class. The usage of AI in education also show the principle of virtue. For example, AI is helpful in screening students whose family are unable to afford their education by collecting information about students’ daily expenditure and then offering them financial assistance in a less obvious way, which will protect their face. AI can also be used to designed special functions for disabled students to make their learning easier.Secondly, in my opinion, the principles of ethics are also practiced in the application of AI in education, which consists of transparency, privacy, justice, fairness, equity, non-maleficence and responsibility. For the principle of transparency, teachers can use artificial to ascertain the learning situation of every student and improve his or her teaching scheme based on that. For example, in my sophomore year, my teachers often used yuketang, an online teaching platform, to assign homework and tests. That platform will analyse the basic situation of everyone for teachers automatically, like strengths, weaknesses and progress. It actually proved effective in practice. Some educators use AI to collect data about students’ working situations after graduation and see whether their students need any help from school and government. In this way, students can obtain employment chances from more various channels, which will help them to find a job more suitable. The use of AI to ensure justice and fairness is very extensive. Now there are many websites and platforms where you upload your intellectual achievements like papers, and you can get a guarantee that it is originated by you and any else won’t be able to steal it. As far as I am concerned, AI plays a vital role in protecting educational equity. With the e

My Views on the Essence and Principles of Ethics of Artificial Intelligence Used in Education

With the rapid development of science and technology, artificial intelligence came into being and became more and more prosperous. In our daily life, we can find the application of this technology everywhere. Recently, it has been largely used in education, which played an important role in teaching and learning. Under such a background, it is crucial for teachers and learners to know the essence and principles of ethics of artificial intelligence used in education. The reasons for that can be drawn from the following two perspectives.

First of all, only when we have a better understanding of the essence of something can we use it in a more appropriate way. Utilitarianism, as the first point of the essence, emphasizes the function of artificial intelligence. In the modern classroom, multimedia became an indispensable tool for teachers especially for foreign language teachers. Using a power point which displays the new vocabularies and grammar points, students can have a clear image of the knowledge they will learn. Besides, the teacher can show pictures and videos easily to attract students’ interests. The application of artificial intelligence in class brings not only convenience for teachers but also better performance of students. This let people see the utilitarian side of this new technology. As for the deontology, the application of artificial intelligence care more about the feelings of learners. With more and more courses can be learned online, students can set their own paces for learning. This is quite different from the traditional class, which students with poorer learning foundation have no way to relearn the knowledge that they did not understand in class. Besides the advantages for teaching and learning, the morality of humans, virtue is also emphasized in the essence of the application of artificial intelligence in education. The three essence works together to help people know better the artificial intelligence in education. Therefore, if we want to make full use of artificial intelligence in education, the first thing is to make clear what the essence it is of this technology in this specific area.

Secondly, the principles also can help people know how to use artificial intelligence in education properly. As a relatively new technology, artificial intelligence has not been strictly regulated by the law. This provides chances for some people to applicate it illegally, such as stealing the personal information of students and changing the grades of examinations. One of the reasons for those phenomena is the ignorance of the principles of artificial intelligence used in education. In the area of education, the majority is immature students, so it is important to protect their privacy. In addition, the most important meaning of the application of a new technology in education is to ensure the fairness of rights to be educated of every person.

In conclusion, from my point of view, it is crucial for teachers and leaners to know the essence and principles of artificial intelligence used in education if they want to use this technology in a more appropriate way.

On essence and principles of ethics in AI

The 21st century has witnessed a significant development of artificial intelligence (AI), at the meantime some aspects about it have caught public’s eyeballs. As to the essence of AI used in education, there are mainly three components, they are utilitarianism, deontology and virtue. They are the things we must bear in mind when we use AI in education, or we will make some disastrous impact in our world.

Utilitarianism suggests that the reason for our frequent usage of AI in education is that we need it to make our life easier, as long as we can benefit from it then it is meaningful. It focused on what one thing can give us, whether it can help us in many aspects, whether it is useful for us. Usually, the more useful things are given more importance, while the useless things are ignored. For us, AI is an important tool to deal with many problems in our life, with it many impossible things have become possible. It’s normal for us to take utilitarianism into consideration, but focus too much on them may not be practical. During our interaction with the AI, we’d better seek a balance point.

Different from the previous one, as a system of ethics, deontology not focus on the utility of AI, but aims at regulate the means we use AI. It has a clear-cut boundary between right and wrong, with this principle it constrains our behaviors with AI. It judges the correctness through the user’s intention and his behavior, if the user is trying to use it to give more fun to students and achieve a better result of the class. And he has did it with acceptable behavior without harming other’s benefits, then no matter what has happened resulted from his behaviors he has no fault. This principle concentrates more on behavior than on the result.

Virtue is another aspect of the ethics, it gives prominence to morality and character. Compared with the deontology, it doesn’t focus on the consequence of the behaviors, instead it mainly highlights one’s morals and ethics. Thus, people who are noble, or of high moral can make use of the AI better since they can make true and virtuous reactions facing difficulties or temptations in the process.

Transparency, privacy, justice, fairness, equity, non-maleficence, responsibility comprise the principles of ethics, together they can guide our usage of the AI in education.

When we use AI, we should also provide appropriate transparency to the public, to let more people get familiar with AI and promote the popularity of AI in education. But we should also protect our own profit by sticking to the boundary of security. We human beings should advance together, but privacy is of great importance for individual. We should not only protect our own privacy, but also avoid to invade other’s privacy. Justice, fairness and equity are also applicable in principles of ethics, these principles cannot be violated in any field, or the world will be in chaos.

Non-maleficence indicates that no violence is allowed in AI, it should be a peaceful and profitable field, not a slaughter. Responsibility makes it clear that everyone using AI should take on his own responsibility, only in this way can we make a breakthrough in many troublesome problems.

Enjoying the proficiency provided by AI in education, in turn we should comply with the principles of ethics in AI used in education. With the proper use of AI, a series of breakthroughs in education will take place.

The Essence and Principles of Ethic of Artificial Intelligence Used in Education

With the popularization of the notion of the meta-verse, more and more artificial intelligence has been applied in education. Diverging from robotics-assisted teaching to immersed environment learning, artificial intelligence has been providing background migration and real-scene experience across time and space to students, which has artificially extend students’ ability and fully explore human intelligence.

Considering artificial intelligence used in education, some of its limitations should be weighed more. Such as how to weigh the essence of artificial intelligence used in education and principles of ethics, I think the principles of ethics are the baseline of artificial intelligence, which means these principles should be based to large extent. However, the transparency, privacy, justice cannot always be ensured when utility of artificial intelligence being regarded as the primary function.

First and foremost, deontologism is the basis of principle, the criterion and the cornerstone. In a socialist society, it is essential for the artificial intelligence used in education to meet the practical principle. Artificial intelligence used should be based on respecting personal freedom and safety, which means for most of time, humanity and human rights outweigh the egalitarian use of artificial intelligence. Deontologism, which can also be interpreted as socialist humanism, suggesting that human is the focusing point, and more attention should be paid to human behaviors and thoughts, not simply deteriorate human to a tool of machine. What’s more, considering from deontological perspective, personal interests should not outweighed over the public interest.

Secondly, the transparency, privacy, justice cannot always be ensured when utility of artificial intelligence being regarded as the prim function. Artificial intelligence serves for students. What can be done is to broaden their horizons and improve learning performance, such as the rise of the meta-verse to provide students with background migration and real scene experience beyond time and space limitation. However, the notion of intelligence means that non-human interference, which accompanied by numerous unconscious machine processing. During the process, without human interference, the indifference of machine characteristics might be exposed, and unethical problems, such as privacy invasion, maleficence actions might be conducted by artificial intelligence unconsciously. The algorithm interference suggests it is difficult to avoid injustice, unfairness, inequity incidents. In intelligent era, algorithms also understand humans more than humans themselves, and how to guarantee privacy and avoid transparency really need further consideration.

Kindness is a good quality of a person to regulate and restrain themselves, but not everyone has acquired this good quality. The applying of kindness varies from person to person, it is also not subject to law. Ethical guidelines are an invisible benchmark for AI in the process of serving people. But transparency, privacy, fairness and justice cannot always be ensured when utility of artificial intelligence being regarded as the primary function.

When applying artificial intelligence in education, what should be noticed is that human is the focusing point, and more attention should be paid to human behaviors and thoughts, not simply deteriorate human to a tool of machine. When considering the utility of artificial intelligence, the transparency, privacy, justice should also be taken into consideration, without human interference, the unethical problems of machine characteristics might be exposed. The algorithm interference suggests it is difficult to avoid injustice, unfairness, inequity incidents. In intelligent era, more attention should be paid to algorithms. Only in this way can artificial intelligence be better served for education.

As a postgraduate and a potential teacher, I am supposed to have an understanding of artificial intelligence in education from the point of essence and principles.

The first essence of AI is utilitarianism, which means AI is a good helper. For one thing, It is no doubt that with the use of AI, classes can be more interesting. Teachers are able to show various pictures and videos that are closely related to learning materials, which on the one hand makes students pay more attention to the class, on the other hand increases the efficiency in their study. For another thing, with the prevalence of artificial intelligence, students can be easily accessible to a large amount of online resources. Students will not be bothered because of limited studying materials written in the heavy books. In this way, study becomes much more convenient and strength-saving.

The second essence is deontology. We have to admit that although artificial intelligence brings we humankind a lot of advantages, it also has drawbacks given that some people may make use of this technology for personal gains and they may hurt others in order to get what they want. It is the same in the education, for example, some people may steal others’ academic achievements and trade illegally with the support of AI technology. Therefore, while enjoying the convenience brought by artificial intelligence, we should ask ourselves to abide by morality and follow the third essence, virtue. Insisting virtue requires people engaged in education to respect others’ results and to do good things for people around including our teachers, peers, and students with the help of artificial intelligence.

In addition, principles of ethics of artificial intelligence in education deserves interpretation.

The first is transparency, which is conflicted with accuracy to some extent. It is difficult for scholars to have transparency and accuracy at the same time while we are doing researches about education. That is to say that if one wants research results to be more transparent, then the accuracy will be decreased accordingly.

The second is privacy. Artificial intelligence always triggers problems about privacy because it involves data. For instance, people need to fill in private information online in order to sign in some apps, the information includes name, contact manner and educational background. Some education institutions may steal relevant data and get in touch with you and recommend educational products that matches your educational level, such as courses.

The third is justice, fairness and equity. This means that people from different social background are equal in front of artificial intelligence, and no one should be discriminated against. In the educational field, students and teachers are supposed to en joy the equal opportunities to be accessible to the resources without considering their region, religion, race, gender, etc.

The fourth is non-maleficence, which indicates that artificial intelligence is supposed to be used for the benefit and well-being of human beings, instead of harming us. This requires us to use and develop this technology rationally and properly. For example, we can use it to improve the efficiency in study, but we cannot let it control our thinking.

Last principle is responsibility. Everyone engaged in education has the responsibility to take the consequences caused by what they have done by using artificial intelligence. Problems cannot always be perfectly avoided given that artificial intelligence is still in the way of developing. Thus, people should be careful, prudent, and also responsible.

In the future, artificial intelligence will be undoubtedly used more widely in the education, but the essence and principles will always lead us.

Recent decades have been witnessing an unprecedented application of artificial intelligence (AI) in numerous areas, education in particular. AI-aided education, with great convenience and utilitarianism, has stormed into every quarter of pedagogical circle and it becomes much more prevalent t under the context of COVID-19 pandemic in that online courses are in dire demand. The fact that AI can greatly facilitate both learning and teaching in education naturally raise a question of the essence and principles of ethics of AI, a question that most people don’t ever bother having a shot at thinking or asking when they indulge in the kaleidoscopic world of AI flourish.

From the perspective of human-orientation, I contend that the essence of AI, to a large extent, lies in its utilitarianism, especially on the wings of sharing economy. As a beneficiary of AI, now I am taking a final exam using online questionnaire called “Sojump”. Admittedly, having recourse to AI, the examinees don’t bother to bring a pen and paper and the results was instantly submitted and saved, sparing the teacher the trouble of identifying the fuzzy handwriting. It is in this sense that the integration of AI and technology is a bless to all people and making both teaching and learning much more easier in that learner can secure a sea of information whenever and whatever they want employing such technologies as Rain classroom, Wechat, Kohoot!

My highlight on the practical aspect of AI doesn’t mean that I deny essential fact that AI is also making a difference in other perspectives, say, deontology and virtue, which, in nature, are in dialectical complementation with utilitarianism. When more and more learners are becoming blessed with the potent vehicles AI offered, a convey of deontology and virtue is made possible. In poverty-stricken area, learners can have an easy access to various online courses as many as the learners in advanced and prosperous area can get, which will stand the chance of changing their whole life.

Nevertheless, in the course of enjoying the enormous benefits AI provided, people diverged greatly in viewing the principle of ethics including information transparency, user’s privacy, justice, fairness, equity, non-maleficence and responsibility. As I see it, the advantage of AI-aided education overweigh its disadvantages. With proper regulation, transparency can be appropriately controlled in case the information is not evenly allotted to every learner. In the era of information boom, it is imperative for every learner to have a good capacity of obtaining the information he wanted and always be wary of latent perils which will undermine privacy. As education is becoming more and more accessible with the aid of AI, the tricky questions of education equity and fairness is being addressed gradually in that AI technology can provides poor children better education, the only thing they need is if a phone and the internet. In addition, non-maleficence is best mirrored in the online learning and teaching since the lack of face-to-face communication, which is a great relief for introvert learners who dare not answer questions in the presence of others and learners who resist from any advanced technologies newly occurred. In the right path of AI development, every “traveler” bears its responsibility, heavy or light, to make the education circle a better place, a small deed such as not intruding others privacy and making every attempt to invent a software, will make all the difference.

Artificial Intelligence Used in Education Need to be Perfected

With the development of science and technology, more and more advanced technologies have been widely applied in the field of education, which promotes the improvement of educational level, but it also leads us to think carefully about the essence and principles of ethics of artificial intelligence used in education. We need to think of it dialectically. Some aspects may be favorable, but others are not.

As to the essence of artificial intelligence, it includes three aspects, that is, utilitarianism, deontology and virtue. Firstly, Artificial intelligence is certainly a technology with utilitarianism, it makes machines help teachers deal with some tasks in education, It can not only improve efficiency and free teachers from busy assignments, but also take care of each student in the same way, so that teachers can concentrate on their own teaching and research, students can constantly correct their own mistakes and improve themselves. Secondly, when we talk about the deontology of artificial intelligence, we tend to focus on the moral obligation and the duty. We know that artificial intelligence technology is created by men so that machines can replace men to complete some tasks, but they can't be exactly the same as human beings, for example, they don’t have thinking ability, morality. They can only follow the program to perform tasks, they do not know what is right and wrong when encountering emergency. Lastly, it actually makes us think about its contributions to virtues. The application of artificial intelligence in education itself is to promote the development of education and even further promote the development of mankind.

As to principles of ethics of artificial intelligence used in education, we also need to think about transparency, privacy, justice, fairness, equity, non-maleficence and responsibility. About transparency, artificial intelligence used in education can ensure transparency, we can keep track of what students are learning, how are they getting on with their learning and then make teaching adjustments according to their situation.

And about privacy, each student's learning is only known to him and his teacher, which to some extent ensures each student's privacy. The next three principles of ethics, that is, justice, fairness and equity, all of them pay attention to the fair treatment of people, when artificial intelligence used in education, it can ensure fairness, machines are different from men, machines will only in accordance with the preset procedures and standards to complete the corresponding tasks, they treat every student in the same way. About non-maleficence, because machines don’t have emotions or feelings, so even if students have some excessive or inappropriate behaviors, machines will not be angry, and will not say or do something harmful to the students. They are still going to follow the instructions as usual. So it can be said that to some certain extent, it can avoid teachers' impulsive behaviors that bring harm to students. Lastly, about responsibility, artificial intelligence technology applied to education is highly responsible in the sense that it follows one set of tasks after another with little or no mistakes. On the other hand, it doesn't go beyond what it's told or programmed.

In general, artificial intelligence used in education have some good aspects in essence and principles of ethics. In the future, the application of artificial intelligence in the field of education will become a trend. Therefore, it is necessary to further improve the shortcomings of artificial intelligence technology in order to better serve education field.

The Essence and Principles of Ethics Should Be Observed When AI Is Engaged in Education

Artificial Intelligence (AI) has gained much prominence in recent years, and it has also been used in a variety of sectors, such as logistics, communications, and finance. While in the field of education, AI is still on its way to efficiently benefit both teachers and students. Currently, AI has been utilized in various courses such as general education, physical education, computational education, etc.

It should be admitted that much convenience has been brought into our daily life by AI. However, as a newly-born technology, it still has some issues in terms of ethics. Three salient ethical issues remain to be addressed: how to protect users’ private information? who should be responsible if the information leak? And how to make sure the fair use of AI technology in areas with different economic levels? To tackle these complications, before related and useful regulations and laws come into effect, the solution is to observe the essence and principles of ethics.

To solve the first problem, the essence of virtue and the principle of privacy and no-maleficence in ethics should be followed. As a popular saying goes, everyone is running naked in the era of big data. It also applies to the era of AI. When AI is introduced in the field of education, the basic information of students and teachers such as gender, age, face, fingerprint, phone number, and so on will be collected for later use. In addition to the advanced technology of the protection system itself, the aforementioned essence and principles are the last psychological firewall to safeguard individuals’ privacy.

Concerning who should be in charge of information leaks, the principle of responsibility is supposed to be taken into consideration. Recent years have witnessed many incidents of information leakage and trading, like the information leakage of Facebook in 2018, causing psychological trauma to the public and negative effects on society. Education is the base of the country, providing intellectuals for future construction. If the AI is applied in education, related organizations like schools, universities, and the Ministry of Education should be responsible for the safety of users’ information and eliminate information trade.

As for the fair use of AI technology in areas with different economic levels, this tough problem calls for the observation of justice, fairness, and equity principles of ethics. It is well known that the use of AI technology such as in distance education requires both software like high-speed Internet and hardware like laptops, tablets, and smartphones. These requirements can be easily achieved in economically developed areas but may be impossible for students and teachers in poverty-stricken regions. As a consequence, the application of AI technology in education might broaden the gap of educational inequities. Taking this dilemma into account, the principles of ethics above should be obeyed. An alternative way may be piloting in economically developed areas first and giving necessary help to poor districts.

In conclusion, when AI is adopted in the field of education, the essence, and principles of ethics much be followed before related policies are in effect to tackle the three issues, namely, the way to safeguard private information, the responsible organization to deal with information misuse and the fair use of AI technology.

Essence and principles of ethics of artificial intelligence in education

The 21^st^ century has witnessed considerable development of science and technology. Artificial intelligence has sprung up as an unprecedented force which has been applied to the educational field. Technically, the emergence and application of artificial intelligence should considerably advance the cause of education. However, in the case of being used wrongly, artificial intelligence can be very dangerous. Therefore, some vital factors are needed to be considered while using it, which are the essence and principles of ethics.

The essence of artificial intelligence has several elements including utilitarianism, deontology and virtue. The creation of a novel technology is bound to benefit people with its unique utilization values. With artificial intelligence, our education has an opportunity to proceed in a multi-dimensional way. Teaching/learning through videos, pictures, games etc. is accessible, which has greatly developed our education. Artificial intelligence has influenced we human beings more than ever. Under this circumstances, we have to attach great importance to it’s deontology. Artificial intelligence should be used and analyzed without causing any harm to human beings. Besides, virtue is another crucial factor of artificial intelligence that we can’t neglect.

The principles of ethics include transparency, privacy, justice, fairness, equity, non-maleficence, responsibility and so on. Artificial intelligence is a double-edged sword. In order to get rid of the detrimental sides of the artificial intelligence, we must pay attention to the principles of ethics. First of all, the most important policy concerning to our education system is justice and fairness. Our government has made a lot of efforts to promote equity in education to make sure that everyone including those from big cities and villages and from different nationalities is accessible to equitable educational resources. As an important tool of education, artificial intelligence has no other choice but to undertake this task. Second of all, in the process of teaching/learning through artificial intelligence, all steps and functions have to be designed and programmed under the guidance of the principle of privacy. Privacy is a big issue for the age of “big data” with all the data being connected and intertwined worldwide. And personal information leakage is an important source of fraud and other malignant network of crime. So if the students’ personal information and data are leaked, the consequences would be unimaginable. And besides, transparency is also a factor that we can’t neglect. Artificial intelligence should be working in a transparent way. Last but not least, artificial intelligence should be used under the guidance of responsibility. Programmers and those who use it should all take the responsibilities. This is an era where everyone has their own microphones so many different voices are interacted around us. But in terms of education, we need to be responsible for what we say and what we do to let artificial intelligence provide the most values.

With all that being said, artificial intelligence should be applied to education under these principles of ethics. Every coin has two sides, artificial intelligence is no exception. We need to pay attention to them while using it in education.

Artificial intelligence, as we all know, has penetrated many fields of human life. As to its advantages and disadvantages, it varies from people to people, and it also varies from one field to another. Artificial intelligence has great potential in education in the future, to a certain degree, which will help teachers impart knowledge to students more effectively. However, artificial intelligence in education is still a controversial topic.

Which essence of artificial intelligence, you may wonder, is acceptable in education? First of all, from the perspective of utilitarianism, students can grasp what they learn more effectively through using artificial intelligence, which is helpful for our teaching program. For example, we can apply personalized learning to students, that is to say, that each student will have their own learning plan particularly designed for them. After all, every student has different characteristics and what they are good at is also various. Moreover, as time goes by, according to the change of their learning path or rhythm, personalized learning can also adjust their learning plan to make the most accurate change. Secondly, as to the deontology, in my opinion, it is also acceptable. Because artificial intelligence can never replace teachers, and this kind of teaching make students learn more interestingly. Thirdly, the virtue of artificial intelligence is what people talks most frequently. If it is useless to our education, I think they will not apply it to this field. As I mentioned before, the utilitarianism is the virtue of it.

There are still many principles of ethics of artificial intelligence that we should pay attention to, such as transparency, justice, non-maleficence and responsibility. To begin with, it may let out students’ privacy, because artificial intelligence is all about the statistics and website, which also makes students’ learning be transparent. Everyone may obtain the information of students if there is no the protection. Secondly, the use of artificial intelligence in education is also injustice. Unlike students in urban, it is hardly possible for students in the rural or even more distant area to share this learning way, which will cause the bigger gap between students’ learning. Thirdly, it might do the harm to student’s psychological health by making them under pressure. Using artificial intelligence in education may increase the learning path, which may cause the anxiety of parents and students. Last but not least, we do not know who will be responsible to it if there are something wrong with artificial intelligence.

All in all, as to the artificial intelligence used in education, in my view, it will be promising as long as people can use it properly. But in the process of development, People should take all the essence and principles of ethics into consideration. Why we apply artificial intelligence to our education, because we are willing to make our education better by using new methods. But if it cannot make it, it is unnecessary to use it in our education. Not just in education, we should use it properly in every field.

My opinion on Artificial Intelligence

With the development of science and technology, more and more novel instruments are introduced into classrooms. At present, the most popular and influential instrument is artificial intelligence.

Science and technology is a double-edged sword. That is to say, they can be useful tools, but when abused they can also be a stumbling block to human progress.

On the one hand, the application of artificial intelligence in education can be beneficial and useful. That is to say, the use of artificial intelligence in education follows utilitarianism. This point of view is not groundless, it is a theoretical point of view supported by scholars. For example, Mayer has put forward Multimedia learning theory, Paivio has also proposed the dual-coding theory. Many scholars have also carried out experimental studies. When picture, video and audio are used in teaching, students' learning effects are improved. Of course, the application of Artificial intelligence is not limited to pictures and videos, it's much broader, online education, study applications, flipped class and so on are all included. During the Covid-19, online education has greatly improved teaching efficiency and solved the problem of studying at home without going out to school. The application of artificial intelligence can activate the classroom atmosphere, mobilize the enthusiasm of students, reduce the workload of teachers, and provide a learning platform for some students. In this sense, AI is a powerful and useful instrument.

Despite the advantages of artificial intelligence, the ethical principle of artificial intelligence should not be ignored. As education becomes more and more intelligent, the role of teachers in teaching is gradually weakened. No matter how advanced the technology is, artificial intelligence cannot replace teachers. Artificial intelligence is unemotional, but teachers are affinity and infectious people, teachers’ every word and gestures will have a greater impact on students. If teachers just abuse artificial intelligence and teach mechanically without emotion, students' experience and gain in learning will also reduced. If teachers are no longer responsible for their teaching, so how can we develop students' sense of responsibility?

At the same time, artificial intelligence also has an impact on the principle of responsibility. Although artificial intelligence has brought convenience to learning, will this convenience have a negative impact? At present, many translation applications and learning applications are widely used in students' learning. If students can make reasonable use of these auxiliary devices, it will have a positive effect on learning. However, if they search on the Internet as soon as they have to do a homework or use translation applications as soon as they write an essay, it will seriously affect students' independent thinking ability.

With the development of modern artificial intelligence, many people no longer abide by the principle of fairness and justice, and academic misconduct such as plagiarism is increasing. This abuse of science and technology has not brought progress to our education, but is a retrogression of human morality and ethics. In the face of convenient technology, some people lose the virtue of honesty and trustworthiness for utilitarianism.

Technology is supposed to promote human progress, artificial intelligence is supposed to be a useful instrument in teaching and learning. In the process of using artificial intelligence in the future, we should abide by the principle of ethics, follow the principles of fairness and justice, responsibility and non-maleficence, so that human virtue can continue.

Artificial Intelligence in Education

Recent years have witnessed the prosperous development of artificial intelligence in education. Particularly, after the outbreak of Covid-19, online learning further strengthens this need. Considering the irresistible trend of applying artificial intelligence to pedagogy, we may think about some questions: will artificial intelligence replace teachers? Does it only exert a positive effect on learning outcomes? The answer is definitely not, because every coin has two sides, and artificial intelligence is not an exception. Therefore, we have to set some limitations to maximize its advantages and rein its potential harms.

The essences of artificial intelligence reflect our concerns and requests about it: Utilitarianism, Deontology, and Virtue. First, humans are quite pragmatic. We will never create or even talk about useless things. The reason why we apply artificial intelligence in education is that artificial intelligence can satisfy our needs to achieve high-quality teaching results. For example, artificial intelligence in math class can be used to draw abstract three-dimensional graphics, which may be difficult for students to imagine. Technologies such as Kahoot are designed to stimulate students’ learning motivation. However, on the other side, if we don’t set application restrictions on the transformational generation grammar as Chomsky did at the beginning, its application will produce many semantically unaccepted sentences, and the same is true of artificial intelligence. We can’t let its powerful power go unchecked, because sometimes it will hurt human beings. Artificial intelligence in education should always focus on serving teachers’ teaching and students’ learning. It can not infringe human rights and go against the wishes of users. This also implies another essence of artificial intelligence, that is, artificial intelligence technology should not do harm to its users, it should have virtue. For example, in education, artificial intelligence should stimulate students’ desire to learn, help students grasp knowledge, and achieve better learning outcomes. These essences reflect the original intention of scientists in designing artificial intelligence: it should promote the quality of life and advance educational progress, rather than go against the common will of mankind.

The essences of artificial intelligence determine the principles it should obey: transparency, privacy, justice, responsibility, non-maleficence, and so on. These principles rely on each other. Justice means that students of different learning levels, gender differences, and from different countries and regions should be equal facing AI. For example, by analyzing the learning results of English by German and Chinese sophomores through artificial intelligence, it is often possible that the results of German students are better than Chinese students, however, can we say that Chinese students are not as good as German students? Definitely, the answer is no. German and English belong to the same language family, therefore, German students may learn English more quickly. If we ignore the relevant cultural background and then use artificial intelligence for data analysis, it is quite unfair.

Another principle is privacy. How to ensure that AI can keep a balance between obtaining enough information to boost education but not infringing on users’ privacy is a key point for us to consider. For example, some learning software may require students’ voices, face recognition, personal information, etc., but the disclosure of this privacy may bring challenges to users’ information security. In order to better comply with the above principles, we require higher transparency of artificial intelligence in education, and we need to set up a strict accountability system for artificial intelligence so that it can always serve the cause of human education, not run counter to it. Therefore, we should always uphold the original intention of the application of artificial intelligence in education.

The issues of ethics of artificial intelligence used in education

With the rapid development of technology in modern society, artificial intelligence has been applied in quantities of fields. In recent years, artificial intelligence used in education has caught much attention of scholars. Classroom based on artificial intelligence has greatly changed the teaching mode of traditional classrooms. Every coin has two sides. Artificial intelligence used in education has its own merits and demerits. It also brings about some challenges to ethics.

Artificial intelligence in education promotes learning effects of students in many aspects. It is beneficial to teachers and students. Students can gain much more information than before easily. They become more interested in learning. Teachers won't feel stressed any more. Artificial intelligence can take place of them to do some repetitive work, such as revises lots of homework and corrects papers. Based on the big data analysis, artificial intelligence can present the weaknesses of every student. For utilitarianism, artificial intelligence provides much convenience for education. However, some issues of ethics about it need to be considered.

There is always a heated discussion about whether artificial intelligence can replace the role of teachers. Some people begin to worry about the career of teachers. As far as I'm concerned, there is no need to be concerned about that. Artificial intelligence can't definitely replace the role of teachers. Artificial intelligence is the machine without emotions. It is made of lots of unaffectionate programmes. It can only provide knowledge to students. As we all know, education is the process of nurturing people. The responsibility of teachers is to impart knowledge and cultivate people. Artificial intelligence can't teach moral values to students. What's worse, overuse of artificial intelligence is harmful to mentality of students. Students will become more and more dependent on the artificial intelligence, which is adverse to develop the critical thinking and innovative awareness of students. Artificial intelligence can also cause threat of privacy to people. We can't live without the internet nowadays. Our all information is stored in smart devices. Once it is leaked to the criminals, our safety of financial properties will face great threat. Beyond that, artificial intelligence may cause injustice, inequity and maleficent in education. Artificial intelligence can't teach students in accordance of their aptitude, let alone their emotional needs.There is also another question. There still exists big gap between the developed and underdeveloped regions. So it is difficult to achieve educational justice all over the country.

We had better take some measures to avoid such a series of issues of artificial intelligence used in education. We must combine artificial intelligence with traditional teaching methods in education. We shouldn't rely on too much artificial intelligence. Besides, the relevant technicians are supposed to improve the procedures of artificial intelligence and make it more suitable for education, especially in mental field. The governments also set up a series of laws to regulate the use of artificial intelligence in education. We should also pay more attention to the problem of privacy. Only doing this can we build up a better education system.

With the development of AI, it is increasingly applied in education. The combination of artificial intelligence and education is triggering a revolution in the field of education. It has been changing the ecology of education, the environment of education, the way of education, the mode of education management, the relationship between teachers and students and so on. Therefore, it is important for educators to fully understand the essence and principles of ethics of "artificial intelligence + education".

When it comes to the essence, we have to consider that it is practical and humanitarian. For teachers, artificial intelligence has become a powerful assistant. Based on big data and artificial intelligence technology, teachers can design scientific and reasonable schemes for students' personalized learning before class. Then artificial intelligence can be used to monitor students' learning at any time. It also can help teachers do some mechanical work, such as marking homework, so as to reduce the burden on teachers. At the same time, AI can evaluate whether students master the knowledge. According to these refined feedback, teachers can adjust teaching progress and teaching content.

As for its principles, it is applied in different aspects. With the popularization of artificial intelligence, educational equity has been realized to a certain extent. In areas with backward education level, online learning can be carried out through free open classes, such as MOOC. It helps students to expand their horizons and they can learn something that can no be taught in the traditional class. Meanwhile, artificial intelligence pays more attention to protecting students' privacy. Students' grades and learning conditions have been effectively protected, which can avoid that students feel inferior when they are compared with others to a certain extent. As for parents, they are responsible for kids’ education. So, artificial intelligence can help them reduce their burden. AI will form a database according to children's knowledge mastery, learning behavior track and multi-dimensional information during and after class by monitoring children's learning progress and concentration. They can understand what their children have master and provide strong support for their children's later learning .

However," Every coin has its two sides". AI also brings about many disadvantages. Firstly, the fact is that artificial intelligence can provide students with individualized extracurricular tutoring, which is based on the premise of the corresponding economic strength. If their parents can not afford the online lessons, they will not have the opportunities to know more knowledge. It is unfair. Secondly, in the field of education, although technology improves the speed and capacity of knowledge, enriches the means of dissemination, and enables information and knowledge to be transmitted to a wider group without boundary, time limit and capacity, but the amount and speed of acceptance of these information and knowledge are limited. When students have online class, they may not be completely engrossed in the lessons because there are too many distractions around students. Also, the phenomenon of involution arises. Many elementary school students are learning programming such as Python and learn extra courses so that they can compete with others.

Someone says that the essence of education is to teach students to be emotional, but the machine is ruthless. Therefore, in my opinion, the best way of education is to combine the AI and the traditional way.

Recent several years have seen incredible improvements in the field of artificial intelligence, which has found its way into numerous aspects of our lives, delivering a heavy influence to everything from economics to daily lives. But the most notable aspect is that on education. However, teaching using artificial intelligence has drawn mixed feelings. Some hail it as a leap for human society, like online learning using computers without teachers’ involvement under the covid-19 pandemic, while others fret that it might be incomplete so that students’ learning outcomes are unsatisfactory. This essay will mainly take an utilitarian look at the online learning without teachers from different perspectives.

Online learning without teachers’ involvement benefited from the virus and was rapidly used around the world. In 2021, we are experiencing the post covid-19 pandemic time, it is necessary to analyze the effects and problems of online learning without teachers’ involvement from the perspectives of students (all levels), and parents. Through reviewing previous works and researches about it, this essay will highlight the students’ perspective from external factors (technical issues) and such internal factors as learning efficiency and motivation. The internal factor will directly affect the learning outcomes of students. In addition, the parents’ satisfaction to online learning will be analyzed and it is related to the students’ learning competency and assignments, which also greatly influence learning outcomes of students.

Traditional teaching method has been gradually replaced by online learning owing to the widespread of covid-19 around the world. For students, the technical issues are the first to be solved. They need modern facilities such as computer, laptop, smartphone and so on, licensed APP, sound internet connection. Some researches have shown that students from impoverished or immature areas such as underdeveloped countries and rural areas lack sound internet facilities and high-speed connection. Whereas students’ online learning in developed areas are successful relatively in this aspect. Even online learning can be regarded as saving and spanning time and space of learning. In this way, it is actually unfair for students in different areas in this aspects. In the traditional classes, face-to-face communication, real-time interaction, body language, facial expression of teachers can be made full use of to give an authentic and effective experience for students. However, online learning is relatively virtual. Most students, researches showed, thought they can’t participate in the classes actively due to the lack of face-to-face communication with teachers as well as the eye-to-eye discussion with class fellows which is conducive to the group working. Those activities are frequent in the traditional classes. That led the poor learning efficiency of students resulting in the bad learning outcomes. In a word, the lack of sociality significantly weaken the motivation of students. However, online learning is beneficial to those who don’t like social contact, particularly high level students, and the protection to their privacy make them feel a sense of satisfaction and security in classes.

The emergence of covid-19 forced some students to learn online at their home. Parents’ satisfaction has also become a point to be focused on, in particular for the primary students. Researches showed that parents’ satisfaction is related to the students’ self-management and teaching design such as the learning time and tasks. Between two factors, the influence of students’ self-regulation is more obvious, the more sufficient competencies of self-management students have, the more satisfied parents are.

The findings can give rise to the enlightenment that good learning outcomes are the results of parents’ moderate involvement as well as the students’ competencies of self-regulation for online learning without teachers’ involvement. We must look at artificial intelligence on education from different perspectives so that we can draw a more pragmatic conclusion and make the further educational optimization.

With the development of technology, artificial intelligence (AI) has been widely used in education. The application of AI in education has been influenced how students learn new knowledge, how teachers impart knowledge and how to access learning outcomes. AI has optimized each aspect of teaching and learning, enriched students’ learning experience, aroused their interest in learning and improved their learning effect. The application of AI helps teachers to unload and give more chances for students to learning. However, there are disadvantages of the AI’s application in education. Students can get answers if they upload the pictures of the exercises in some APP. This will make students lazy and unwell to think. Therefore, this paper will analyze the application of AI in education from different aspects.

In terms of essence of AI used in education, the application of AI in education is very useful and are friendly to all people. AI has changed and promoted the way of students learn new knowledge. Teachers could only teach the same things to everyone at school, but students are not the same. With the help of AI, students could get personal learning plans and they don’t have to learn everything. They only need to learn the unfamiliar knowledge and do the exercises that are not easy for them. In this way, all the students could have their own learning plans, and learning efficiency will be improved. Moreover, AI could help teachers to assess the pronunciation of a language. Teachers have different standards to evaluate and are easy to get tired, but AI can’t be tired. Nowadays, especially during the pandemic, there are plenty of intelligent learning approaches which are free to all people.

While, for some principles of ethics, AI used in education cannot meet all the criteria. AI has not been used in everywhere of the world. It is unfair to the areas with backward economy and science technology. When AI is being used in economically developed places, there are still a lot of students who are learning in a traditional way. Compared with AI teaching, traditional teaching methods are less efficient, less interesting and cannot be personalized. Therefore, the emergence of AI causes unfair education. Some learning resources are publicly available, but there are also AI teaching platforms that require payment. In addition, all kinds of APPs are collecting students' data, and their privacy is not well protected.

In terms of responsibility, the use of AI relieves some of the burden on teachers in lesson preparation and teaching, and also makes students’ learning more freely. Theoretically, one of the responsibilities of a teacher is to pay attention to the behaviors of students. However, some teachers may rely much on artificial intelligence replace their own role of teaching, which not only leads to low learning efficiency of students without human supervision, but also is not conducive to the establishment of teachers' influence. AI can help teaching, but it should not be an excuse for teachers to shirk their responsibilities.

In short, the emergence of AI used in education has greatly improved the learning efficiency of students. Students have more interest in learning, so students can better participate in learning. And most AI applications are free for everyone. Therefore, in terms of essence, the application of AI in education is very useful and are friendly to all people. However, the attribution of responsibility is difficult to confirm, and fairness and privacy are not protected.

The modern world has been witnessing dramatic development of artificial intelligence used in education over the past decades. Recently more scholars begin to have an acute awareness that artificial intelligence may tumble somewhere.

Artificial intelligence, in its essence, is the use of computer technology to make machines think and do things as what human beings do. In terms of its utility, artificial intelligence helps a lot such as in establishing personal plans for each student or checking assignments through Big Data technology. Space-free and time-free study can also be a sparkling point of artificial intelligence. At first glance, AI gains much attention for its high-efficiency, convenience, and intelligence as the term shows, but its seemingly promising future market has ignored a bigger picture.

As obvious as its advantages, the bane of AI can hide nowhere. From my perspective, artificial intelligence has a long way to go especially in terms of privacy, responsibility and emotional focus.

The first and foremost risk in artificial intelligence used in education is the violation of privacy. When enjoying the product of AI and admiring its high-efficiency, the users, either teachers or students, can not avoid of being exposed in such a Big Data era. Today we gradually become accustomed to the shocking words from the other end of the telephone, where you can hear your academic grades, social media accounts and even purchasing bills of educational courses.

Another aspect we need to concentrate on in education area is the emotional attention of students. AI has created some digital devices, which are quite useful in promoting teachers’ teaching procedure. Through artificial intelligence, teachers can deliver tasks, check assignments and establish personal schedules for students of different academic levels. More importantly, there is no longer the limitation of time and space which puzzles the traditional classroom teaching. Distance education makes it possible for students and teachers exchange information through online platforms during the COVID-19 pandemic. However, no matter how much convenience AI has brought to us, a key point in education is gaining less attention, that is students’ emotion care. Through the inanimate machines, students emotional need can not be satisfied. In addition, there is less and less student-student or teacher-student interaction.

The final bane can be captured in the question “ Who will take the responsibility in some AI issues?”. Some accidental events related to artificial intelligence have not yet earned a satisfactory solution. Take traffic accident for example, we have more or less heard the news that someone had been hit by a driverless car. In such a case, people no longer talk about the benefits the artificial intelligence. Instead, what they pay more attention to is that who is responsible for the accident, the driver or the car manufacturer. Then back to the educational area. Some people predict that machine translation will in one day replace human translation. As the trend develops, more and more talents become aware of the fact that they will lose their jobs someday. As a consequence, those talents are less willing to devote themselves to education course. But who can take the responsibility of this trend? If no, artificial intelligence should be improved in some aspects.

From its essence, AI is used to help we human beings through some computer technologies. As a result, some improvements in the ethic aspect should be made in artificial intelligence.

The Use of Artificial Intelligence in Education: Bone or Bane?

In the modern times of the 21st century, the use of artificial intelligence in education has witnessed a significant increase. Basically speaking, the use of AI in education is a strategy that is invented by some scientists who aim to increase efficiency and reduce the workload of our human beings. As to this phenomenon, some people who are strong utilitarians contend that it benefits their life a lot. Nonetheless, opponents argue that it may invade their privacy. Therefore, I believe that this phenomenon is worthwhile to discuss. As a student who cherishes the ability of critical thinking, I am greatly convinced that it is of vital significance that the use of AI in education, a two-edged sword, should be treated prudently for the reason that although it can generate enormous benefits, it may also lead to tremendously terrible catastrophes.

On the one hand, the use of artificial intelligence in education can save as a bone for our human beings. First and foremost, it can reduce our workload since our human beings don’t have to bother doing tedious work. By using AI, this kind of work can be done effectively. In addition, the use of AI in education, which is still a new approach for students who have been taught under the guidance of traditional approaches for many years, can pique their interest in the process of acquiring knowledge. Thus, their learning outcomes can be upgraded. Moreover, the use of AI in education can also be a supporter for parents. In this modern century, the knowledge that the students obtain is rather complicated, and it is merely impossible for parents to understand them and assist their children when they encounter some difficulties in completing their homework. But if the parents can have a good grasp of how to manipulate the simple AI devices, it will become much easier for them to help their children in learning.

On the other hand, the use of artificial intelligence may have the potential to invade the privacy of the users. Since artificial intelligence devices are so intelligent that they know the preferences of the users very well. These brilliant devices can offer the things that they like and filter the things they dislike by simply watching which buttons they click. The users’ personal information is thus stolen. Furthermore, the excessive use of artificial intelligence can lead to the reduced responsibility of the teachers. Since the artificial intelligence devices are so intelligent that many teachers may tend to overly depend on them. They may not be that serious in planning their teaching tasks and just let the artificial intelligence devices teach students. The teaching outcomes can be reduced due to their unserious attitude.

In a nutshell, it is difficult to say whether the use of artificial intelligence is a bone or bane for our human beings since the use of it is a mixture that processes both advantages and disadvantages. Our human beings ought to make full use of its benefits and try our best to fight against its disadvantages in the process of using it.

Nowadays, artificial intelligence is ubiquitously used in education and is effective in all kinds of education fields. Although it brings many positive influences, it also can trigger off some unpleasant outcomes. As a result, it’s important to pay attention to the essence and principles of ethics of artificial intelligence.

As for the essence of artificial intelligence used in education, the first is utilitarianism which means that the products of artificial intelligence should be useful whenever and whatever. It should function as a helpful instrument without resulting in reductant effort. It can save time for teaching and studying and it’s easy to implement among all kinds of educative contexts. Furthermore, deontology and virtue is more about morality. The artificial intelligence in education should take care of students’ self-esteem, satisfaction and cognitive ability who are the main roles in education. Deontology requires artificial intelligence used in education should be appropriate to students and pose no psychological burden on students. Besides, it should be legal and has been tested. Virtue means it should be more humanized and tolerant to the students. It can encourage the students to study and stimulate their intention. For example, it can be seen that students can get some gratitudes and cheerful feedback after doing their homework on line.

The principles of ethics include transparency, privacy, justice, fairness, equity, non-maleficence and responsibility. The artificial intelligence used in education can be judged by public and should protect the private information of the users. Justice means it is designed be certain individual or group without plagiarizing others. Fairness and equity means every learner and every teacher would enjoy the same right and no privilege is involved. Non-maleficence means it should be gentle and not brings about extensive negative outcomes. Probably some studying applications would not threaten the e-products such as phones and ipads. At last, if the artificial intelligence used in education needs amendments, certain individuals should take the responsibility and improve it according to the possible feedback. For example, all the e-platforms allow people to complain.

From my perspective, the reason why the artificial intelligence should meet all the essence and principles is as follows. Students’ self-esteem and satisfaction plays an important role in education. if their esteem is threatened or they has no interested in that artificial intelligence, they will not want to collaborate which does great harm to their learning outcomes. If the artificial intelligence used in education is not utilitarian, student would spend more time than traditional education and it increases cognitive loads. Besides, it’s also inconvenient for teachers. Positive feedback would encourage students’ pleasure to study.

Artificial intelligence used in education would engender many negative influences without following the principles pf ethics. The e-platforms may in a mess because it’s hard to say whether it is effective or not. Some individuals would design it out of economic benefits instead of educational benefits. Furthermore, students would like learn from others, they would not want to be discriminated so it’s important to keep equity. Responsibility prevents individuals abusing the use of artificial intelligence.

To conclude, only by following the essence and the principles of ethics, can artificial intelligence use in education make great importance in improving students’ learning outcomes, saving teachers’ efforts and improving the artificial intelligence itself.

High technology is transforming our lives dramatically, changing the ways in which we work, study and even live. With the development of computer science, artificial intelligence(AI)

is undergoing unprecedented growth and being applied in many other fields including environment, transportation, medicine, culture and education.

Nowadays, AI is widely used in education such as intelligent tutors and online classroom. It enables teaching and learning more convenient and diverse. At the same time it is necessary to have a clear recognition of the essence and principles of ethics of AI. I think AI is a tool used by people so first of all the artificial intelligence used in education should be utilitarian. It mainly includes two parts. On the one hand, it should be easy to handle. Online tools or applications is used by students and teachers of all age groups. So if the operation is too complicated it practicability will be reduced . On the other hand, educational technology should also continue to develop to meet the needs of teaching and learning.

In my opinion, there are three principles of ethics of artificial intelligence. The firstly one is that we should realize that AI could not replace teachers. For a long time to come, artificial intelligence will only assist in the education. Only teachers can imperceptibly affect children and help them form positive values. Secondly, as for students they should know that artificial intelligence changes learning patterns, but not the goal of study. Artificial intelligence can lower the threshold for learners to access knowledge and broaden the way way for acquiring knowledge. But as learners, we should be aware that artificial intelligence is only a technology, and its role is to assist rather than replace. Artificial intelligence can provide ideas but can not help learners solve the problem.

At last, when carrying out artificial intelligence education, we must pay attention to the ethic issues. We should fully consider the new role played by artificial intelligence in educational activities and be careful of the risks bring about AI. We should not take advantage of artificial intelligence to make profits for individual or harm the interest of others. For example, from the perspective of students, if some original works or papers are produced entirely by the artificial intelligence, it may cause complex ethical questions. From the perspective of teacher, in the process of using educational artificial intelligence to collect data it may involve personal safety ethics. Therefore, it is necessary to explain the ethical security risks behind it to teachers.

All in all, we should also understand the limitations, uncertainty and risks of artificial intelligence and learn to use technology correctly. For example, when artificial intelligence help education, the technology it relies on may have a certain degree of unreliability, resulting in mistakes in analysis. As teachers, we should help students identify unreasonable content and information in time and filter them. As students we should also have the ability to choose appropriate courses. So teachers and students can cooperate to establish a healthy educational environment.

With the development of the technology, artificial intelligence has gained rapidly popularity in all respects. It is also widely used in the scope of education. Some people hold the view that artificial intelligence used in education may involve a series of problems, such as individual privacy, equity and justice. While others may insist that any institution have responsibility to ensure this basic principle and it’s artificial intelligence that human beings have opportunity to advance. I would hold the middle of the road position, which takes advantages and weaknesses for us.

When we think of the artificial intelligence, the word ‘powerful’ maybe the first one to come to our mind. As we all know, it has many a advantages. Nowadays, humanity apply it in education because it has potential to address some of the biggest challenges. As the outbreak of the corona virus, people may risk their lives studying in the class. Innovate learning meets the needs for those people, it provides an opportunity for people to study online.

From the aspect of deontology, artificial intelligence contribute a lot in education. When people fall ill, they may resort to it to study or work online. With this methodology, students will also achieve efficacy at the same time. It’s convenient for us to study online, which we can study at any time and in everywhere whenever it’s comfortable. We won’t be confined to study in the classroom.

While it also inevitably brings certain risks for our lives, we can’t turn a blind eye to its disadvantages. When we collect individual information for scientific research, as a result individual privacy may in danger. Because we need quite a large number of messages to ensure the reliability and validity of research done by us. People have reason to doubt the individual privacy. They may fear that individual information will be leaked out and whether strangers will protect their privacy.

Artificial intelligence may also bring harmful effects to us in education. People dedicate their time to doing research may get fewer points than those plagiarizing other people’s research. In this respects, more and more students will doubt the fairness of the artificial intelligence. Truly, it has both advantages and downsides for us in our daily lives. It aims to achieve a shared understanding of the opportunities and challenges that artificial intelligence offers for education.

While people doing research, they must hold the viewpoint that they bear certain responsibility in their mind. We should have a sense of responsibility in the process of research. Because we should be as rigorous as possible in the field of the research. In the future, our research results will contribute a lot to our society.

From the foregoing saying, we can draw the conclusion that artificial intelligence has gained increasing popularity in education. It brings advantages and potential risks for our lives, which so far have outpaced debate nowadays. In my opinion, artificial intelligence has more benefits compared to the disadvantages it brings to us in education. Artificial intelligence is changing the way we think, live, learn and so on.

With the rapid development of modern technology and artificial intelligence, a lot of new educational approaches and forms have now been applied in education. The application of AI technology has received both positive and negative feedback. Then, people start to wonder what is the essence and principles of ethics of artificial intelligence used in education.

First of all, the essence of AI technology generally refers to utilitarianism, deontology and virtue. Utilitarianism means that technologies should be developed and utilized for the well-being of human beings to the uttermost. Then, deontology and virtue generally emphasize a certain kind of relationship between people and technologies. To be more specific, deontology means that people should be consistent with the essence that we should treat AI technologies just as they are human beings and give AI technologies the kindness and virtue we give to human beings.

The principles of AI technology normally include transparency, privacy, justice, fairness, equity, non-maleficence and responsibility. When applying AI technologies to education, we should first of all make sure that the whole process is transparent, which means that both the teachers and students should be well informed of the current situation and all the information appearing in the class. In researching and developing AI technologies, we should enhance security and transparency of artificial intelligence system, and realize supervision, predictability and reliability. Besides, we should also avoid prejudice and discrimination. Then, the importance of privacy is also self-evident. The class is an open place where teachers and students share common information together. In this situation, both teachers and students should be aware that we should try to protect and respect each other’s private information. The development of artificial intelligence should respect and protect individual privacy and fully protect individual's right to know and make choices. Specific principles and norms shall be established in the collection and use of personal information. Besides, justice is another key aspect when using AI technologies in education. Justice and fairness mean the quality of being fair or reasonable. Therefore, in AI assisted classes, it is important that both AI technologies and humans should be treated in a fair and justified way. Then it comes to equity. Equity refers to a situation in which everyone is treated equally. Therefore, in AI assisted classes, both artificial intelligence and students should enjoy the same rights and same status. Non-maleficence is another important principle in AI class. As the basic principle of bioethics, non-maleficence is regarded as the core principle of applied ethics. Finally, responsibility means the duty one shoulders when performing a certain task. In AI class, both the AI technologies, teachers and students have their own role and their own responsibilities.

To sum up, various activities of artificial intelligence should promote human well-being and facilitate human-computer harmony and friendship. At the same time, we should promote fairness, justice, and equal opportunities. We should better coordinate the relationship between artificial intelligence and human beings to ensure that artificial intelligence is safe and reliable. In AI classes, the programmers, teachers and students should work together to achieve the best learning effects. In the future, we will have a better prospect in developing and applying AI technologies in education.

In this intellectualized age, artificial intelligence is applied in human society, which enormously promoted the effectiveness of work and quality of life. Meanwhile, artificial intelligence has also been used in education, and has the tendency that the application will be more widely used in the future. Then, the whole society begins to focus on the essence and ethical principles of artificial intelligence used in education. From my perspectives, artificial intelligence is utilitarian, which must be widely used in educational area, but it can only be used to aid human being, rather than replacing human being, given that it is ill at taking responsibility.

Artificial intelligence must be widely used in educational area. Firstly, teachers have the limitation of taking good care of all the students in the class, but artificial intelligence could make up the limitation. For example, when ten students all want to ask a teacher ten different questions, it would be impossible to answer those questions in a short time. And when a teacher teaches more than one class, it would be a huge amount of homework to correct. This is when teachers need artificial intelligence. Artificial intelligence would answer students’ question immediately, if the answer cannot help them, then they can ask their teachers for help. And for homework, instead of taking hours to correct, artificial intelligence would correct them at once. Secondly, artificial intelligence would improve the justice of education. In society, the educational equality is always a tough issue. For one thing, educational resources are highly centralized in regions with developed economy. For another, cheating occurs frequently in all kinds of examinations. These are the problems that artificial intelligence can solve. Students in under-developed regions can listen to the class given by excellent teachers online, and for those who cheat in examinations, artificial intelligence could recognize them indirectly. Thus, artificial intelligence has high utilitarianism and can improve educational justice. It must be widely used in education.

Although artificial intelligence needs to be widely used, it still cannot replace teachers. Artificial intelligence is cold and mechanical, which cannot give students emotional support and comfort as human teachers. Teachers do not only impart knowledge and wisdom. More importantly, they help students with their psychological problems and tell students the ethical standards, which cannot be done by artificial intelligence. Furthermore, if artificial intelligence is overly used in education, it would make the education losing its initial intention. For example, a student become problematic, who drinks wine all day, or fight with other students. If there is no human teacher to talk to him, only a machine, the machine may choose to give up on this student, given that the machine does not have any emotions, not educating him and helping him. Then, this student may become a criminal in the future, which is against the initial intention of education that guiding students to become ethical citizens. Artificial intelligence may have benefits in imparting knowledge and promoting justice, but it is ill at educating students psychologically. Thus, it cannot full take the responsibility of teaching.

Therefore, in educational area, artificial intelligence can only be used to aid human teachers, to promote the justice of education and effectiveness of teaching. It cannot overly used in education or, in other words, replacing human teachers. In conclusion, artificial intelligence used in education has more utilitarianism, but it lacks of morality.

AI, the Panacea of Education?

Education is the process of teaching and the acquisition of knowledge and values. Teachers can be the educators and learners themselves can also teach themselves. Today’s education has witnessed a vast change in the tools and mediums of teaching and learning. Nowadays, the artificial intelligence serves the process of teaching and learning. It seems that the balance between educators and learners has been destroyed by the “man-made product”. Living in such a society calling for morality and benefits, artificial intelligence should find its path to humanity, benefits of learning and most importantly, the maximum effect of the combination of these factors.

As artificial intelligence is the opposite side of natural intelligence, it is displayed by high-tech machines. Many people doubt that how machines are able to serve as a teacher--- the carpenter of soul. Our natural human teacher seems to be the better choice. Is that really so? Let’s talk about the utility of artificial intelligence first. Our technology today is developed enough to shape the well-being of AI, our machine teacher. Human engineer has filled the AI with all the knowledge concerned with each subject so it is available to teach the learner every solution to the mathematical problem or how long the Renaissance had last. As a saying goes, An AI teacher can take the place of seven human teachers. In a word, AI is economically friendly.

It seems that AI is the panacea and encyclopedia in our process of learning. But now, think of the famous Trolley Problem, what will be the answer that our AI teacher tell us? According to Deontology, the [morality](https://en.jinzhao.wiki/wiki/Morality) of an action should be based on whether that action itself is right or wrong under a series of rules, rather than based on the consequences of the action. Human ourselves still have lots of problems concerned with morality and machines are still cold. How can such morality framework and virtue of human set up in our subordinate product? It is still confusing.

What’s more, as for the result, learners may achieve success in the exam after the teaching of AI. We should look back on the process of learning and teaching. Generality of AI is what we need to consider about. Not every learner is able to afford AI in terms of money, time and space. If 36 students of 40 in a class are able to learn with the aid of AI and they get great success in the exam, the result seems to be positive. Those four who can’t work with AI are left behind. We may draw a conclusion that there is positive correlation between AI teaching and acquisition of learning. Then we are caught into the trap of Deontology. It also disobeys the principle of transparency and fairness. Living in such an era of information, what learners do will be seen by the AI. It is the monitor of our life sometimes. To avoid the leaking of information calls for more actions. So, we should pay more attention to these issues in the application of AI. In the view of Utilitarianism, the application of AI should maximize the well-being of affected individuals and its utility. These conform with the issues mentioned above.

Keeping balance between benefits and well-being in the process of education, especially the one with the aid of AI, matters a lot. I hope the further study can show us the reasonable solution to the issues about learners’ learning and what the better way is for human educator to go along.

The emergence of artificial intelligence (AI) heralds the new era of the world. It exerts a great impact on many aspects of human life, including education. Since AI is so new a thing which appears in the world, for a time someone thinks highly of it while others question it. In the following part, in terms of the essence and principles of ethics of artificial intelligence used in education, I will share some opinions.

Primarily, in the aspect of essence, AI is boon whether for teachers or students. As for teachers, AI can reduce their workload, and they can therefore have more time to makes plans of their classes. We know that insufficient allocation of educational resources in our country is a problem to solve. In some schools, one teacher might have to deal with the studies of dozens of students. While with the help of AI, before the one on one tutoring between teachers and students, AI can alleviate the problem by virtue of solving common problems of students. Thus, on the one hand, teachers can spare more time to make adjustments of their teaching plans to improve teaching quality; on the other hand, they can cope with the particular problems of the particular students.

As to students, AI is good tool to help them with their study. First, for some students who still feel difficult to communicate with their teachers freely, AI is a suitable partner. Take English learning as an example. With regard to spelling or pronunciation, students who are afraid of making mistakes in front of teachers may feel easy to use AI. Moreover, AI can teach students in accordance with their aptitude. So AI also reduces the workload of some students to some extent.

Secondly, apropos of principles of ethics of AI, AI can facilitate the justice, fairness and equity of education. On the one hand, for some students in remote mountainous area, teachers may fail to meet standard teaching standards. By AI, students there can finish their learning online. On the other hand, in terms of handicapped or special students, it may be difficult for them to study like typical students in school. With resorting to AI, AI can help convert speech into text or sign language and employs various ways to teach them. AI makes a contribution to human society. In this way, we can say that AI realize the principle responsibility.

While in terms of transparency and privacy of the application of AI in education, there might still exist risks and challenges. International society calls for transparency and privacy of AI. That is to say, the information and the algorithm of artificial intelligence should be transparent, but it may be a challenge for it to stay private. For instance, in the area of medical care, in universities or hospitals, students may resort to the collected data related to human bodies, and these data may be extremely confident. In such condition, if people still require the algorithm to stay transparent, it may create opportunities for illegal attacks by hackers, let alone the realization of non-maleficence.

To sum up, as far as I am concerned, AI is a double-edged sword: it can only realize the essence and principles of ethics partly. In the long run, it still needs developing and completing.

Artificial intelligence in education

For hundreds of years, the traditional and dominant format for conveying instructional messages has been through oral and written speech in the form of face-to-face lectures or books. However, the rampant Covid-19 was assessed by the WHO as a global pandemic that showed person-to-person transmission and social distance was required between people in order to curb dissemination of disease. Therefore, online learning attracts more and more attention and includes many advantages,such as remote learning,comfort and accessibility. Particularly, the advent of computer technology, especially computer graphics, animation, and interactive visualization technologies, further promoted visual ways of representing information. Artificial intelligence, one of the most advanced technology, enjoys the highest popularity in current researches, including the educational fields.

Artificial intelligence refers to an area of computer science that makes it possible for machines to copy intelligent human behavior. Furthermore, some scientists try their best to develop machines that can think on their own, acquire novel knowledge and have emotions like humankind. Artificial intelligence has been used in various fields. For example, fingerprint and face recognition are safer and more convenient if we want to unlock something without the passwords. What’s of great importance is that AI like some robots can also do things that are too difficult or dangerous for people to do. From the perspective, It is universally acknowledged that AI plays an important role in our daily lives.

In the educational field, scientists also regard AI as a advanced and practical tool for the development of education. AI can be applied to online learning which can enable pedagogical agent to have human intelligence. Social cues, such as expressions, gestures and eye gaze, can facilitate learners' performance and satisfaction. Human intelligence can help the agents to achieve them. In addition, AI can upgrade the educational environments by providing advanced technology.

On the other hand, the development of AI remains controversial because scientists aims to develop AI until it have consciousness like human. Nowadays, some labs have succeed in achieving the goal. The phenomenon seems to be a threat for human teachers whose jobs will be replaced. The entire educational system may be forced to change in order to cater the radical trends of revolution and traditional face to face instruction will disappear completely. In fact, teachers can gain sufficient and elaborate feedback from students which seemed as the most efficient way to judge whether their teacher program and pace is appropriate for each individual student during face to face teaching. In addition, the development of AI need a lot of investments, so the application to all students around the world has a long way to go. Therefore, fairness of education may be threatened. Furthermore, once the machines with artificial intelligence have their own consciousness, they may lose human's control, which may resulted negative influence. Particularly, if some researches take advantage of the powerful of AI to crime, which will be a significant challenge to morality and laws.

Generally speaking, there is no doubt that AI makes a big difference to our life in some ways, but it, as a new technology, also has some aspects to consider. Only when we make good use of it,can it bring us great benefits.

My View on AI Used in Education from the Perspective of Essence and Principles of Ethics

The recent years have witnessed the widespread use of artificial intelligence in education, especially amid the global landscape of COVID-19 nowadays. Education refers to the process of acquiring knowledge and promoting the well-rounded development of students. Artificial intelligence in education refers to the use of technology in classroom in general and online learning in particular. For example, in China, with the policy of “suspend classes without stopping learning”, online learning has become a normal learning style in the past two years. Students can take lessons through many apps such as ZOOM, Dingding, MOOCS instead of face-to-face learning.

In my view, the use of AI has brought us many benefits but meanwhile many problems in terms of the essence and the principle of ethics. The proper way is to think and use it critically.

For one thing, from the perspective of utilitarianism, AI is useful to learning and teaching, in other words, it extends the ways of learning and teaching. For instance, some researches revealed that online learning can improve students’ academic performance and their motivation. For higher education, through online learning apps such as MOOCS, students can take lessons made my other universities and then combine what they learn online with their own knowledge or they can learn from MOOCS to enrich their prior knowledge. In doing so, students are motivated and perform well in their academic achievement.

For another, the transparency is higher in online learning than in traditional classroom. Through online learning apps, information is shared online and students can get access to what they want. For example, many Chinese students have difficulties in learning English, then they can search information online in that there are many lessons in online learning apps such as TED, Khan, etc. In this way, students can improve their learning efficiency and they don’t needs to learn at a fixed time.

However, AI has many defects in terms of essence and principles of ethics. Although online learning plays a key role, but the role of teacher cannot be replaced. For one thing, the deontology and virtue cannot be improved significantly through online learning, they should be cultivated by teachers and also practiced by students themselves. Although students can take online lessons about virtue and deontology, they are more expected to practice it in their daily life. For another thing, in terms of principles of ethics, privacy is a problem that has drawn increasingly public attention. Due to the shared information online, many scholars don’t obey the academic ethics and copy other researchers’ academic findings. For example, many scholars in higher education as well as some stars such as Zhai Tianlin were exposed to copy others’ papers and then they were punished. They do not take the corresponding responsibility as a qualified scholar. Therefore, in this sense, AI has its deficiencies.

In conclusion, AI in education or online learning has brought us many advantages and disadvantages. We should enjoy the benefits of online learning critically, but at the same we should take some responsibilities in that AI cannot replace teachers in traditional classroom.

Nowadays, the artificial intelligence has emerged in every area of the modern life. There will be an evaluation and consideration of artificial intelligence in terms of utilitarian, equality, privacy as well as morality.

In terms of utilitarian, the appearance of artificial intelligence has greatly improved the efficiency of learning. Firstly, artificial intelligence is customized, which can adjust the content and difficulty of learning in time according to the progress and habits of learners. It make learning more scientific as it pays attention to the individual differences of learners and thus brings efficiency. Secondly, new teaching methods and models are emerged as the technology has advanced. For example, the flipped class is a typical use of the techniques which enables the students to preview the teaching materials through media players, and talk about the questions about the class after their previewing, dealing with some of the problems before class with the help of their classmates through the discussion and cooperation. However, the artificial intelligence fails to pay attention to the emotion of learners, which are impossible to be calculated by a computer.

From the perspective of equality, there are some characteristics of artificial intelligence in that it is a media of sharing educational resources. The teaching methods and ideas provided by artificial intelligence come from the cooperation and research of many experienced educators. Therefore, the educational resources available to all learners using artificial intelligence are abundant and of high quality. The drawbacks of artificial intelligence in terms of equality are also obvious. Students in poor areas do not have the conditions to enjoy the convenience brought by artificial intelligence. They can't even get an Internet connection. As a result, excellent resources are in the hands of students with advanced technology, and the gap between rich and poor will become more obvious in the future.

In terms of privacy, artificial intelligence provides students with privacy, for online learning avoids students' embarrassment or even aversion to learning caused by wrong answers in class. Some learning software provides a platform for students to ask questions and answer questions freely, which makes it possible that some novel ideas may be put forward by students without any burden of being doubted since they can send anonymous information. However, in the era of “big data”, there is a risk of information leakage when students register and log in their accounts in some apps. The "private customization" of artificial intelligence will be more accurate only when it has mastered a lot of students' private information, which may lead to a large number of information leakage. A deluge of advertising and fraud will spring up to gullible students and parents. A typical example is the pretended video call between the students and their parents in order to get “extra tuition fees”.

When it comes to morality, the development of science and technology always brings such problems, as the Gene-edited babies are despised by many parents. Artificial intelligence is no exception. In John Watson's famous plush experiment, the child never gets over the shadow of his infancy in his life. Compared with medical science, the development of artificial intelligence in education has encountered relatively few ethical issues, but the pedagogical theory on which artificial intelligence relies is generated in many experiments similar to Watson's. Nowadays, there are almost no such cruel experiments, but in any research on human psychology and other aspects of human beings, the the subjects should be fully selected and respected. With the development of artificial intelligence becoming more and more mature, it may be possible to use artificial intelligence to simulate the psychological state of learners and the state of human beings in the future to replace human subjects.

To sum up, the development of technology and artificial intelligence is unstoppable. The trend of artificial intelligence is to become more similar to humans. Artificial intelligence in education provides convenience and help for students' learning and teachers' teaching, although some functions and problems still need to be improved.

The Essence and Principles of Ethics of Artificial Intelligence Used in Education

With the development of science and technology, artificial intelligence in used widely in education nowadays. It is recognized as an important way of improving the quality of education. But there are also different says about the essence and principles of ethics of artificial intelligence used in education. The following are the argumentations in detailed ways.

Considering the essence of the ethics of artificial intelligence in education, it can be summarized into one word: utility. Any invention of human beings is for the benefit of human beings. At the same time, it cannot produce the harmful effects for the people. Otherwise, the production of artificial intelligence will be of no significance. As for the application of artificial intelligence, it is considered as the progress of human beings. Firstly, it can save the time of teachers and students. With the development of artificial intelligence, people can do a lot of jobs with less time and great efficiency. For example, with the help of artificial intelligence, people can learn knowledge at any time and in any place. Secondly, it can benefit the people at work who are eager to receive education. For example, it makes long-distance education available. As long-distance education is urgent for people at work, artificial intelligence can enlarge the receivers of long-distance education. Thirdly, it can make the product of science and development be available to people. For example, machine translation is basically accepted by teachers and students in education. Especially the software of translation is a great help for the teachers and students.

However, with the application of artificial intelligence in education, it is of great necessity to outline the principles of ethics of artificial intelligence used in education. Firstly, it should respect the morality of human beings. Any scientific development is achieved for the sake of the wellbeing of human beings. Therefore, the morality is the basic principle. For example, the insulting words and expressions should be avoided in the usage of artificial intelligence. Secondly, the rules and regulations should be made to guarantee the normal progress of artificial intelligence used in education. For example, it should be emphasized that the artificial intelligence should not be used when students are taking exams. Thirdly, artificial intelligence should be controlled and regulated at any time. No matter what kind of invention it is, the commander must be human beings. Otherwise, it will damage the human beings instead of benefiting them. Fourthly, artificial intelligence should be used under the condition of fairness and equality. Mostly, artificial intelligence should be reached at a high price. Government should guarantee the availability of artificial intelligence to the poor. Fifthly, the application of artificial intelligence should avoid the reach of privacy. Any development of science and technology should respect the privacy of people. For example, the private information used in the artificial intelligence should not be leaked out.

In conclusion, artificial intelligence in education is a progress of education, for it has a lot of advantages and benefits. Although the essence of artificial intelligence in education is utility, it should avoid the harmful effects at the same time. Therefore, it should be noticed that rules and regulations should be made to guarantee the normal operation of artificial intelligence in education.

Recent years has witnessed the rapid development and dramatic advantages of artificial intelligence(henceforth AI) in education. The major AI-based devices that have been widely used in education include a Bayesian network model which was designed to detect students’ different learning styles in an Artificial Intelligence Online Course and an intelligent robot “Sage” which was applied at the Carnegie Museum of Natural History to offer educational information to visitors. It’s undoubtedly that AI in education has brought many benefits to students and teachers. This is in accordance with the essence of AI.

The essence of AI in education which includes utilitarianism, deontology and virtue derives from the problems existing in the educational field. For example, though the rapid development of computer science, teachers also need to correct students’ homework one by one, which occupies a large amount of time for teachers. A correctly programmed intelligent robot can solve the problem by sharing the mass task and analyzing students’ achievements and problems. Therefore, teachers will have more time to design more teaching activities and improve the teaching quality according to the results given by the robot. AI can also improve the lack of high-quality teachers and poor teaching quality in poor areas, and promote education equity. Teachers are usually unwilling to go to poor areas because the life there is too tough. Though the government has already offered policies to encourage teachers to teach in poverty-stricken regions, the phenomenon that one teacher takes the responsibility for teaching students of many grades is also common in those regions. With the help of AI, students there can get access to more learning resources, which will certainly promote the education equity and the protection of human rights. But it’s not enough to have good starting points, principles are also needed to restrain the technology to ensure that it will work.

The principles of ethics of AI include transparency, privacy, justice, fairness, equity, non-maleficence and responsibility. When a new AI-based device is designed, all students, teachers and institutions should have the right to get access to it. Technology should not become a method of monopoly or privilege. Only in this way, educational equity will be guaranteed. The privacy means the protection of students’ information. AI-based device will ask for students’ agreement for getting access to their information, sometimes private information so as to assess their learning achievements. Under this circumstance, technicians should make sure that their devices won’t crave users’ personal information. As we have mentioned before, AI can help improve the teaching quality in poor areas. But there is another problem. Due to the unbalanced economic development, the development of teaching hardware and software is unbalanced, and the teaching conditions are uneven. Many poor northwest regions haven’t installed the projectors and other equipment. Multimedia classrooms and computer classrooms are also under construction. When introducing AI into these regions, the existing problems should be tackled first.

In a word, AI will significantly promote the development of education with its positive and meaningful essence. But in order to make the essence come true, AI should also observe the principles of ethics.

The 21st century has witnessed the prosperity of artificial intelligence and its widely usage in various areas. Discussion now centers on whether the application of AI technology in the field of education brings more harm than good. Despite tremendous benefits and convenience it has brought to us, we ought to be cautious of the potential downsides that lurked in the dark. Personally, I’m convinced that the application of artificial intelligence in education should comply to the principles of ethics.

The essence of the application of artificial intelligence in the field of education lies in the efficiency and satisfaction for personalized needs. Nowadays the prevalence of artificial intelligence has greatly changed our learning environments. We enjoy the convenience of the smart classroom and smart library, just to name a few, that provides us. And our learning data could be collected and then analyzed by artificial intelligence to evaluate our current levels and learning abilities so as to offer us targeted learning suggestions and recommendation. Besides, our whole learning process could also be monitored with our permission. The AI technology could know our learning state via our facial expressions then provides us corresponding suggestions— whether we should move on or take a break before further study. In addition, the application of artificial intelligence lessens the burden of teachers in that most of the work that requires teachers to record and evaluate can now completes by technology in an even more efficient way. Take exams for example, most of the work is done by computer now instead of teachers and other teaching staff. And at the same time, it also gives teachers a quick and visual feedback of students’ abilities and progress.

Granted, from the positive perspective, the application of AI in the field of education can enjoy a lot of merits. Still, issues that could be engendered if AI technology is unbounded by those principles. As for privacy, your personal information could be easily leaked out when you typed those information on certain websites or the camera footage could be stolen for illegal usage. In terms of equality and fairness, there will be an unbridgeable gulf between the rich and the poor. Those who can afford AI education enjoys the cutting edge of educational resources while those who cannot do not have a shot.

Nonetheless, the application of artificial intelligence in education is still immature and needs to be explored. AI technology still cannot replace the role of teachers. The indiscriminate use of artificial intelligence could violate the rule of the education development and act counterproductively to the education. Besides, most artificial intelligence focus more on the studies of the students while neglects their all-round abilities and metal and physical health, which are fundamental to their growth.

In conclusion, the application of artificial intelligence functions now as an indispensable part in our society, yet we need also to pay attention to its possible demerits and problems it may pose. The AI technology should be further developed abiding by the education law so as to cater the needs of education and its own development as well.
